# Hiding in the yolk: A unique feature of *Legionella pneumophila* infection of zebrafish

**DOI:** 10.1371/journal.ppat.1011375

**Published:** 2023-05-08

**Authors:** Flávia Viana, Laurent Boucontet, Valerio Laghi, Daniel Schator, Marine Ibranosyan, Sophie Jarraud, Emma Colucci-Guyon, Carmen Buchrieser

**Affiliations:** 1 Institut Pasteur, Université Paris Cité, Université Paris Cité, Biologie des Bactéries Intracellulaires and CNRS UMR 6047, Paris, France; 2 Institut Pasteur, Université Paris Cité, Unité Macrophages et Développement de l’Immunité and CNRS UMR 3738, Paris, France; 3 Sorbonne Université, Collège doctoral, Paris, France; 4 National Reference Centre of *Legionella*, Institute of Infectious Agents, Hospices Civils de Lyon, Lyon, France; 5 Centre International de Recherche en Infectiologie, Université Lyon 1, UMR CNRS 5308, Inserm U1111, ENS de Lyon, Lyon, France; University of Utah, UNITED STATES

## Abstract

The zebrafish has become a powerful model organism to study host-pathogen interactions. Here, we developed a zebrafish model to dissect the innate immune response to *Legionella pneumophila* during infection. We show that *L*. *pneumophila* cause zebrafish larvae death in a dose dependent manner. Additionally, we show that macrophages are the first line of defence and cooperate with neutrophils to clear the infection. Immunocompromised humans have an increased propensity to develop pneumonia, similarly, when either macrophages or neutrophils are depleted, these “immunocompromised” larvae become lethally sensitive to *L*. *pneumophila*. Also, as observed in human infections, the adaptor signalling molecule Myd88 is not required to control disease in the larvae. Furthermore, proinflammatory cytokine genes *il1β* and *tnf-α* were upregulated during infection, recapitulating key immune responses seen in human infection. Strikingly, we uncovered a previously undescribed infection phenotype in zebrafish larvae, whereby bloodborne, wild type *L*. *pneumophila* invade and grow in the larval yolk region, a phenotype not observed with a type IV secretion system deficient mutant that cannot translocate effectors into its host cell. Thus, zebrafish larva represents an innovative *L*. *pneumophila* infection model that mimics important aspects of the human immune response to *L*. *pneumophila* infection and will allow the elucidation of mechanisms by which type IV secretion effectors allow *L*. *pneumophila* to cross host cell membranes and obtain nutrients from nutrient rich environments.

## Author summary

*L*. *pneumophila* is an intracellular pathogen that has co-evolved with aquatic protozoa but can also infect humans to cause a severe pneumonia. *L*. *pneumophila* infections are increasingly recognized worldwide, with outbreaks affecting hundreds of people commonly reported. Although *Legionella* infection has been widely studied in various model organisms, key aspects of human disease and host response to infection are not well reflected in these organisms. Here we have established the zebrafish as a new model for *Legionella* infection and use cutting-edge intravital imaging to reveal the temporal dynamics of bacterial dissemination, and the interactions between bacteria and macrophages/neutrophils, within a whole-of-organism spatial context. We show that as in humans, macrophages are the main drivers of the host defense and “immunocompromised” fish are highly susceptible to infection. In addition, we identified a previously undescribed infection phenotype in which *Legionella* enter and replicate in the yolk region of zebrafish larvae, an aspect which ultimately drives the outcome of infection. The new zebrafish model should prove useful as a platform to study host and bacterial factors underlying *Legionella* infection.

## Introduction

*Legionella pneumophila*, a gram negative, facultative intracellular bacterium inhabits natural, freshwater sources [1,2]. As an environmental aquatic microbe, *L*. *pneumophila* replicates intracellularly in aquatic protozoa [3]. Most interestingly, in contrast to other intracellular pathogens *L*. *pneumophila* is not adapted to a single host, but it exhibits a broad host range including Amoebozoa (amoebae), Percolozoa (excavates) and Ciliophora (ciliated protozoa) [3,4]. In the environment *L*. *pneumophila* can also be found within biofilms or it can survive in a planktonic form for a certain time [5]. As fresh water and engineered systems are connected, *L*. *pneumophila* can also contaminate artificial water systems. Protected within its protozoan hosts *L*. *pneumophila* survives water disinfectants and may infect humans *via* aerosols produced by different engineered structures and devices. The inhalation of *L*. *pneumophila* containing aerosols can cause a severe pneumonia, the so-called Legionnaires’ disease [6]. However, not every infection leads to disease. Disease outcome is determined by virulence of the bacterial strain, the bacterial burden in the inhaled aerosols and most importantly by the host immune status. Host factors determining susceptibility include: age above 50, smoking and/or having chronic lung disease, being immunocompromised and genetic factors that alter the immune response [2,7,8].

Once the bacteria reach the lungs of susceptible individuals, they can infect alveolar macrophages and replicate therein. After being phagocytosed *L*. *pneumophila* avoids lysosomes and establishes an endoplasmic reticulum derived vacuole, named the *Legionella* containing vacuole (LCV) [9,10]. The LCV, a safe haven for bacterial replication, is established by utilizing the Dot/Icm type IV secretion system (T4SS) that injects over 330 proteins into the host cell [9–11]. These effector proteins manipulate a myriad of host pathways such as, recruiting vesicles derived from the endoplasmic reticulum to the LCV, supplying the bacteria with nutrients, restraining autophagy and supressing apoptosis and to subvert the host cell immune response [9–11]. A surprisingly high number of these effectors mimic host proteins and encode eukaryotic functions helping *L*. *pneumophila* to modulate numerous host pathways to its own benefit in remarkably diverse ways [11–13]

Intracellular replication of *L*. *pneumophila* and innate immune responses to this pathogen have been studied *in vitro* using both murine and human cell lines and *in vivo* using different animal models of infection. However, results obtained with these models cannot be easily extrapolated to what is observed in human disease. Studies in invertebrate models, such as *Galleria mellonella* and *Caenorhabditis elegans*, [14,15] require further validation in more developed models as their immune system greatly differs from that of vertebrates. Mouse infection fails to recapitulate the human disease phenotype, as most inbred mice strains are naturally resistant to *L*. *pneumophila*. This natural resistance is due to the activation of the inflammasome through NAIP5/NLRC4, triggered by flagellin, as well as through an apoptosis-associated speck-like protein containing a CARD (ASC)-dependent pathway, resulting in the production of cytokines *via* an IL-1 autocrine loop [16]. Humans lack the NAIP5 allele present in murine cells [17]. The only mouse model able to support *Legionella* growth are A/J mice, as they have a hypermorphic *NAIP5* allele, however knock out mice are rarely available for A/J mice [18]. Very early after the discovery of *L*. *pneumophila*, a guinea pig model of Legionnaires’ disease was developed, as the guinea pig is highly susceptible to *L*. *pneumophila* when infected through injection into the peritoneum [6] or when exposed to *L*. *pneumophila* containing aerosols [6]. Several studies thereafter have shown that the guinea pig infection model recalls human disease and allows to study the immune response to *L*. *pneumophila* infection [19,20]. However, the guinea pig model is rarely used due to the limited availability of specific immunological reagents for these animals and the demanding laboratory and husbandry requirements.

The above-mentioned models, including the widely used murine model, have limitations for studying *L*. *pneumophila* infection *in vivo*. Furthermore, exhibit discrepancies between results obtained in human cells, for example mouse macrophages restrict *L*. *pneumophila* growth *via* caspase 1 and caspase 7 activation, whereas human macrophages do not activate caspase 1 and 7 and thus allow growth of *L*. *pneumophila* [21,22]. Thus, we sought to develop a new, alternative model for *Legionella* infection. The zebrafish *(Danio rerio)* originally introduced as a model organism in developmental biology has emerged in recent years as a powerful non-mammalian model to study nearly every aspect of biology, including immune cell behaviour and host-pathogen interactions [23,24]. Zebrafish are evolutionary closer to humans than fruit flies and nematodes, easier to manipulate than mice and their immune system is remarkably similar to the one of mammals, making them an attractive laboratory model for immunology and infection biology [23,24]. Its popularity is also due to its small size and the natural translucency of its embryos and larvae, which makes it possible to follow leukocyte behaviour and infection onset at the level of the whole organism in real-time and high resolution [25]. Additionally, although adult organisms display a fully developed immune system with both active innate and adaptive branches, studies can also be conducted at the early stages of life (embryonic or larvae) when the organism solely relies on innate immunity, allowing the dissection of mechanisms arising from different immune responses [25–27]. Here we examined whether the zebrafish could be an alternative model for analysing host-pathogen interactions, in particular the innate immune response to *L*. *pneumophila* infection.

We show that *L*. *pneumophila* infection of zebrafish larvae recapitulate human disease onset, as infected wild-type larvae are generally able to clear the infection, but immunocompromised fish fail to do so. Both macrophages and neutrophils quickly interact and engulf injected *L*. *pneumophila*. Macrophage-depleted larvae show a dramatic increase of bacterial burden concomitant with host death, pointing to a crucial role of macrophages in controlling the infection. Interestingly, not all wild-type larvae are able to control the infection; a fraction showed high bacterial burden in the yolk region, a unique zebrafish infection phenotype.

## Results

### *Legionella pneumophila* infection induces mortality in zebrafish larvae in a dose dependent manner

**Fig 1:**
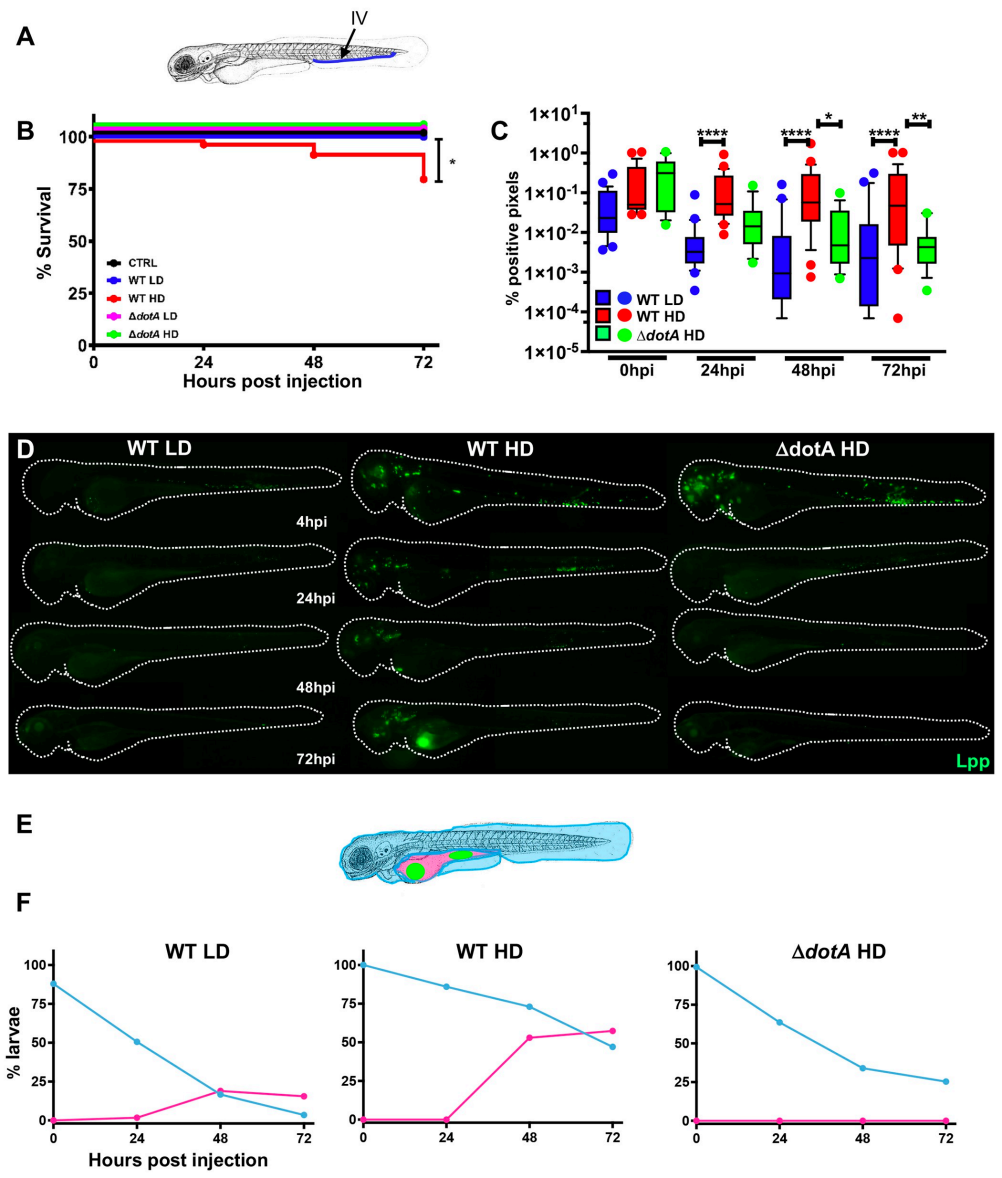
Zebrafish larvae are susceptible to intravenous *L*. *pneumophila* infection in a dose dependent manner. **A)** Scheme of the experimental set up of bacterial infection using zebrafish. A 72hpf zebrafish larva. Bacteria are injected in the bloodstream (iv) via the caudal vein (green arrow). The scheme of the zebrafish larvae has been adapted from [35] and has been previously modified from [87] **B)** Survival curves (three independent experiments pooled) of zebrafish larvae injected with WT-GFP Low Dose (WT LD) (blue curve, n = 60) or High Dose (HD) (red curve, n = 60), or with Δ*dotA*-GFP Low Dose (Δ*dotA* LD) (green curve, n = 12) or High Dose (Δ*dotA* HD) (green curve, n = 36), and incubated at 28°C. Control non-injected fish (CTRL, black curve; n = 72). P < 0.05 was considered statistically significant (symbols: **** P < 0.0001; ***P < 0.001; **P < 0.01; *P < 0.05. **C**) Bacterial burden evaluation by quantification of % of fluorescent pixel counts from images of individual injected larvae followed over time from 0 to 72 hpi. Each larva was imaged daily, and images were analysed with Fiji for bacterial burden quantification. Five experiments have been pooled, for a total of 28 larvae for WT LD and LD, 18 for Δ*dotA* HD. P < 0.05 was considered statistically significant (symbols: **** P < 0.0001; ***P < 0.001; **P < 0.01; *P < 0.05. No symbol on graphs means that not statistically differences were observed. **D)** Representative images of *L*. *pneumophila* dissemination, determined by live imaging using a fluorescence stereomicroscope, of zebrafish AB larvae infected with a LD or a HD of WT-GFP, or a HD of Δ*dotA*-GFP. Individual infected larvae were followed over time by live imaging at 4h, 24h, 48h, and 72h post injection. GFP fluorescence of the injected bacteria is shown. **E)** Scheme of a 72 hpi larva with body (light blue) and yolk (pink) region highlighted. Green dot in the yolk represents bacteria burden. The scheme of the zebrafish larvae has been adapted from [35] and has been previously modified from [87]. **F**) Quantification of bacterial dissemination over time. Larvae injected with LD, HD LD or Δ*dotA* HD were imaged over time, and then scored for the GFP + bacteria absolute presence for each larva over time. Larvae were scored as “infected” when they showed at least one small detectable GFP+ dot. Data obtained were plotted as % of larvae with bacteria in the body (tail, trunk, head; blue curve), or in the yolk (pink curve). 11 independent experiments have been plotted (representing a total of n = 69 WT LD; n = 58 WT HD; n = 54 Δ*dotA* HD infected larvae).

To analyse whether *L*. *pneumophila* can cause disease in zebrafish larvae we microinjected larvae 72 hours post fertilisation (hpf) intravenously in the caudal vessels near the cloaca (Fig 1A), with 10^3^ or 10^4^ CFU of wild type (WT) *L*. *pneumophila* strain Paris expressing GFP (WT-GFP) or the type IV secretion system (T4SS) deficient isogenic mutant expressing GFP (Δ*dotA*-GFP). The infected larvae were kept at 28°C and were monitored regularly until 72 hours post infection (hpi) to record survival or death using a stereomicroscope. Larvae infected with doses of up to 2x10^3^ CFU of WT-GFP (defined as low dose, LD) all survived (100% survival). In contrast, larvae infected intravenously with doses of 10^4^ CFU (defined as high dose, HD) resulted in approximately 30% of death within 72 hpi (Fig 1B). Importantly, all larvae injected with HD of the Δ*dotA*-GFP strain survived for the entire time of observation (Fig 1B) indicating that the T4SS is crucial for replication in zebrafish larvae, as it is in other infection models and in humans.

The progression of the infection was followed by analysing the bacterial load at 0, 24, 48 and 72 hpi comparing three different methods. First, we quantified the pixel counts of GFP fluorescence of live larvae images (S1A Fig), secondly, we analysed the number of GFP expressing bacteria present in lysed infected larvae by flow cytometry (S1B Fig) and thirdly we plated serial dilutions of homogenates of euthanized larvae on BCYE medium (S1C Fig). The results obtained with the three methods showed a similar trend. However, as *Legionella* are slow growing microbes (at least three days to form colonies on plates), they are rapidly overgrown and outcompeted by the zebrafish-associated microbes, even when using selective *Legionella* agar plates, making counting colonies imprecise. We found that measuring the bacterial burden by Flow Cytometry is technically difficult at later time points, as the fish die due to the high bacterial burden and the bacteria are released from the degrading fish. Thus, the bacteria are removed during the washing steps. In contrast, pixel count values only increase when bacteria are alive and divide, thus increasing pixel counts are an excellent proxy for increasing numbers of bacteria and most accurately represent the bacterial load. Thus, we choose to monitor the *L*. *pneumophila* load of zebrafish larvae by fluorescent pixel counts on live injected larvae as the primary method. As shown in Fig 1C, where the bacterial burden was evaluated by fluorescent pixel counts on individual injected larvae followed over 72hpi, most larvae injected with LD of WT-GFP progressively control the bacteria by 24 hpi, with only few larvae showing an increase in bacterial numbers at 72hpi. Similarly, HD of Δ*dotA*-GFP were also progressively controlled by 24 hpi. In contrast, some zebrafish larvae injected with HD of WT-GFP were unable to eliminate the bacteria at 72 hpi, and the bacterial counts remained high (Fig 1C). These results were corroborated by FACS analysis on lysed larvae (S2A Fig). We also monitored infected larvae by fluorescence microscopy. Immediately upon injection (20 min to 2 hpi), bacteria were detectable as small foci, probably associated with professional phagocytes (Fig 1D). By 24 hpi, in both, larvae injected with LD of WT-GFP, as well as larvae injected with HD of the avirulent Δ*dotA*-GFP strain, the GFP signal declined becoming undetectable by 48 hpi, suggesting that the bacteria were progressively cleared. Despite showing the same pattern at 24 hpi, larvae injected with HD of WT-GFP showed an increase in GFP signal at 48 hpi, suggesting that bacterial proliferation occurred in a fraction of the infected larvae. Interestingly, in these larvae, bacterial proliferation occurred mainly in the yolk region where the bacterial foci increased dramatically over time, with concomitant death of the infected larvae by 72 hpi (Fig 1D).

To gain insight into the progression of the infection of LD and HD of WT-GFP or HD of Δ*dotA*-GFP infected larvae, we analysed the bacterial presence in the yolk (pink, Fig 1E), a region where professional phagocytes are unable to enter, and in the rest of the body (tail + trunk + head; blue, Fig 1E), using fluorescence microscopy over time. A single small GFP dot (indicating few bacteria) present in the larvae was scored as positive for infection. We observed that about 60% of larvae injected with HD of WT-GFP and about 20% of larvae injected with LD WT-GFP showed yolk growth at 72 hpi. In contrast, larvae injected with HD of Δ*dotA*-GFP progressively cleared the bacteria, and bacteria were never observed growing in the yolk (Fig 1F). The presence of bacteria in the yolk was intriguing and prompted us to investigate whether this unique feature was dependent on the site of injection of bacteria in the larvae. Thus, we injected 72hpf zebrafish larvae with HD of WT-GFP in different closed cavities such as, the otic vesicle and the hind brain ventricle and compared: larvae survival, bacterial burden, and outcome of bacterial dissemination over time. Only blood stream injected bacteria were found to successfully replicate in zebrafish and establish a proliferative niche in the yolk. This suggests a role of the blood circulation in the capacity of *L*. *pneumophila* to reach the yolk region and to replicate there (S3A–S3C Fig).

Secondly, we tested if a natural route of uptake could cause infection in zebrafish. As in its usual habitat *L*. *pneumophila* lives in freshwater and replicates in protozoan hosts [28], it is possible that fish get infected in the environment by taking up infected amoeba. Indeed, amoebae are prey of zebrafish larvae. To test this hypothesis, we infected *A*. *castellanii* with *L pneumophila* and exposed 120 hpf zebrafish larvae (start of autonomous feeding) to *L*. *pneumophila* infected *L. pneumophila*-infected amoebae and evaluated bacterial survival (S4A Fig) and bacterial dissemination (S4B–S4D Fig) in the larvae over time. While zebrafish larvae engulfed the infected amoebae, as shown by the GFP signal detectable in the exposed larvae at 48 hours post bacterial exposure (S4C Fig), no permanent infection was established and the engulfed bacteria were evacuated with other faecal content without impact on larvae survival, suggesting that this might not be an important route of infection in the environment.

Collectively these results indicate that only bloodstream injected WT *L*. *pneumophila* induce a dose dependent death of zebrafish larvae. Larvae that were unable to control infection by 48 hpi, showed a unique infection phenotype, a high increase of the bacterial burden in the yolk region.

### *Legionella pneumophila* replication in the yolk of zebrafish larvae is T4SS dependent

**Fig 2:**
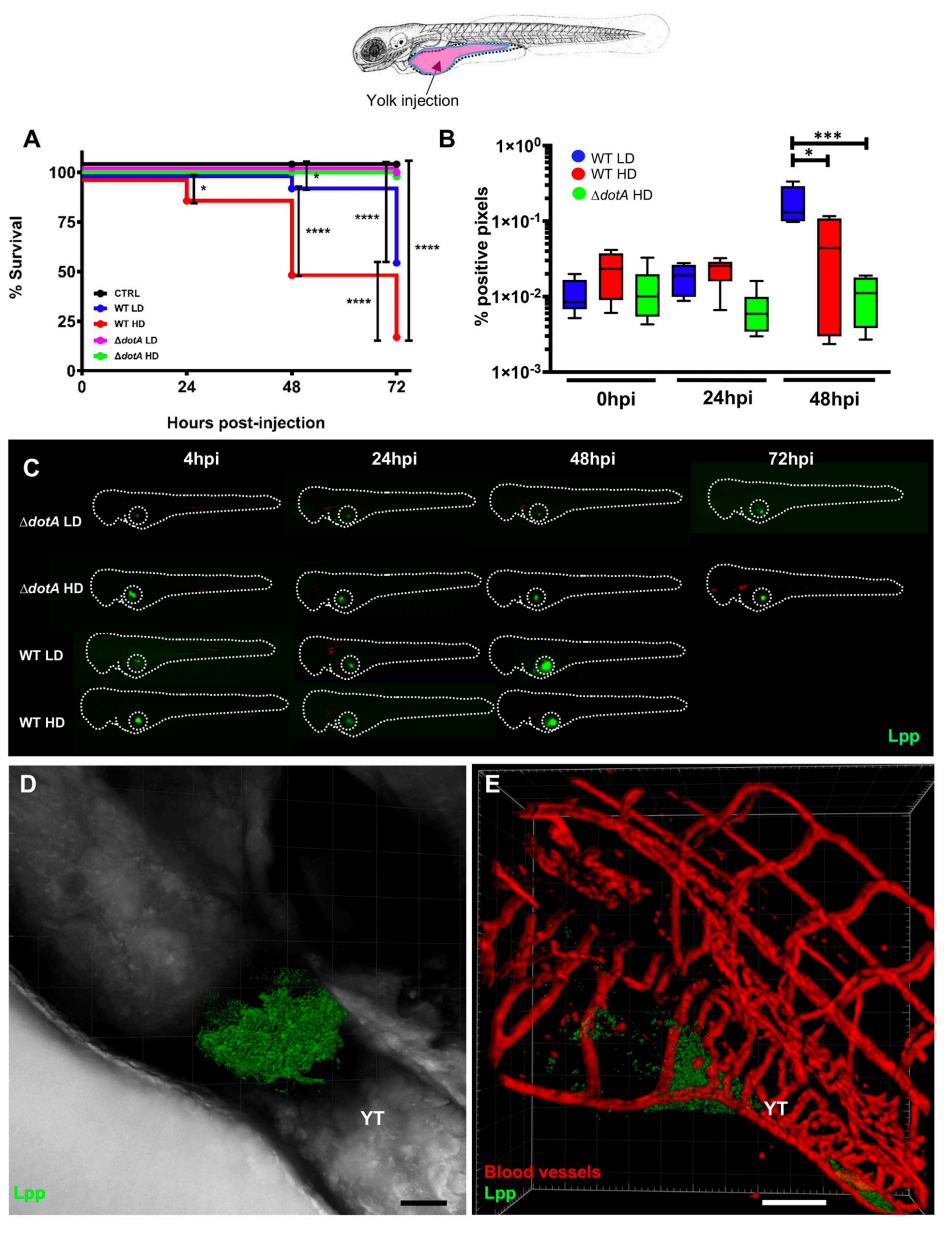
Bloodstream *L*. *pneumophila* establish a proliferative niche in the yolk causing a persistent local infection. **A)** Survival curves (two independent experiments pooled) of zebrafish larvae injected into the yolk with WT-GFP Low Dose (WT LD) (blue curve, n = 48) or High Dose (HD) (red curve, n = 48), or with Δ*dotA*-GFP Low Dose (Δ*dotA* LD) (magenta curve, n = 48) or High Dose (Δ*dotA* HD) (green curve, n = 48), and incubated at 28°C. Non-injected fish (CTRL, black curve; n = 48). The scheme of the zebrafish larvae has been adapted from [35] and has been previously modified from [87]. **B)** Bacterial burden evaluation by quantification of % of fluorescent pixel counts on individual injected larvae followed over time by 0 to 48 hpi. Each larva was imaged daily, and images were analysed with Fiji for bacterial burden evaluation. One experiment plotted, 6 larvae per condition. P < 0.05 was considered statistically significant (symbols: **** P < 0.0001; ***P < 0.001; **P < 0.01; *P < 0.05. No symbol on graphs means that not statistically differences were observed). **C)** Representative images of *L*. *pneumophila* localization, determined by live imaging using a fluorescence stereomicroscope, of zebrafish larvae infected with a LD or a HD of WT-GFP, or a LD or a HD of Δ*dotA*-GFP. Individual infected larvae were followed over time by live imaging at 4h, 24h, 48h, and 72 hpi. GFP fluorescence of the injected bacteria is shown. Dotted circle highlights GFP bacteria in the yolk. **D)** Representative maximum intensity projection of confocal acquisition of a 72 hpi zebrafish larva injected in the bloodstream with HD of WT-GFP, mounted laterally and live imaged using high resolution confocal fluorescence microscope, showing bacteria growing in the yolk and yolk tube (YT) region. The x-y-z raw data were post treated with the LEICA lighting application for reducing noise and processed with Imaris for 3D volume rendering. Related to S1 Movie. **E)** Imaris 3D reconstruction and volume rendering of the *L*. *pneumophila* growth (GFP labelling) in the yolk of *kdrl*:ras-mCherry (red vessels) infected larva at 72hpi, showed laterally. Overlay of GFP and mCherry is shown; BF is shown to help to visualize the yolk region and host anatomy. YT Related to S2 Movie. Scale bar = 50mm.

The replication of *L*. *pneumophila* in the yolk region of infected zebrafish larvae was strictly dependent on a functioning T4SS, as Δ*dotA-*GFP failed to reach the yolk. To investigate whether the secretion mutant can grow in the yolk cell when reaching it, we injected LD and HD of WT-GFP or of Δ*dotA-*GFP *L*. *pneumophila* directly into the yolk cell cytoplasm of 72 hpf zebrafish larvae (Fig 2A). Surprisingly, Δ*dotA*-GFP did not replicate in the yolk even when injected directly, although it persisted over 48 hpi (Figs 2B, 2C and S2B). When LD or HD of WT-GFP was directly injected into the yolk, a higher rate of bacterial proliferation and larvae death was observed compared to the bloodstream injection whereby 100% of the larvae dying have bacteria replicating in the yolk. (Figs 2B, 2C and S2B). These observations suggest that T4SS system is not only crucial for reaching the yolk region but likely that some of its effectors are necessary for the bacteria to obtain nutrients from the yolk environment to allow replication. To further analyse this hypothesis, we selected a mutant in the gene encoding a sphingosine-1 phosphate lyase, (WT, Δ*spl)* [29], as we reasoned that this enzyme might be implicated in degrading sphingolipids present in the yolk of zebrafish larvae and thereby might aid *L*. *pneumophila* to obtain nutrients. Injection of Δ*spl* in the yolk sac region, and analyses of larvae death as compared to WT-GFP or Δ*dotA*-GFP howed that survival of zebrafish larvae injected with the Δ*spl* was slightly but significantly higher than with WT injected larvae (S5A Fig), suggesting that the T4SS effector *Lp*Spl might be implicated in nutrient acquisition in the yolk environment.

Interestingly, the first isolation of *L*. *pneumophila* was achieved by inoculating the yolk region of embryonated eggs probably due to the richness in nutrients provided by the yolk [6]. Thus, we decided to investigate the infection phenotype of *L*. *pneumophila* WT-GFP and Δ*dotA-GFP* in the yolk sac further using as model embryonated chicken eggs (ECE). We inoculated ECE directly in the yolk region with WT-GFP and with the Δ*dotA* -GFP strain at a median quantity of 8.7 log_10_ CFU/mL for each and assessed mortality of the embryos daily (calculated form 4 independent experiments and 42 ECE in total). The survival curves were significantly different (p<0.0001). The total mortality during the 6-day observation period was significantly higher in WT-GFP infected eggs (95%) than in the Δ*dotA-GFP* infected eggs (18%) or PBS inoculated control eggs (24%) (S5C Fig). The highest mortality was observed at 1 and 2 days post infection in WT-GFP inoculated eggs with 79% mortality at day 2 *versus* 18% in Δ*dotA-GFP* or PBS inoculated eggs. Quantification of *L*. *pneumophila* in the yolk sac region at the day of mortality or at day 6 post infection revealed that the number of bacteria in the yolk sac of WT-infected ECE, was significantly higher than that in the yolk sac of those infected with the Δ*dotA*- GFP strain (8.5 log_10_ CFU/mL and 7.4 log_10_ CFU/mL, respectively, p = 0.011) (S5D Fig). Controls inoculated with PBS (n = 4) showed no *L*. *pneumophila* growth. Thus, like in zebrafish larvae only the WT strain can persist and replicate in the yolk region and of inducing mortality in the embryos, while the T4SS mutant strain persists but is not able to replicate and does not induce high embryo mortality. This result further supports the finding that the T4SS is crucial for obtaining nutrients which might be both, proteins and lipids [30].

Taken together, these results suggest that the *L*. *pneumophila* T4SS plays a crucial role for the bacteria to pass from the blood circulation into the yolk and that T4SS effectors play an important role to obtain nutrients for bacterial proliferation in the yolk.

### Bloodstream *L. pneumophila* establishes a proliferative niche in the yolk region causing a persistent infection

**Fig 3:**
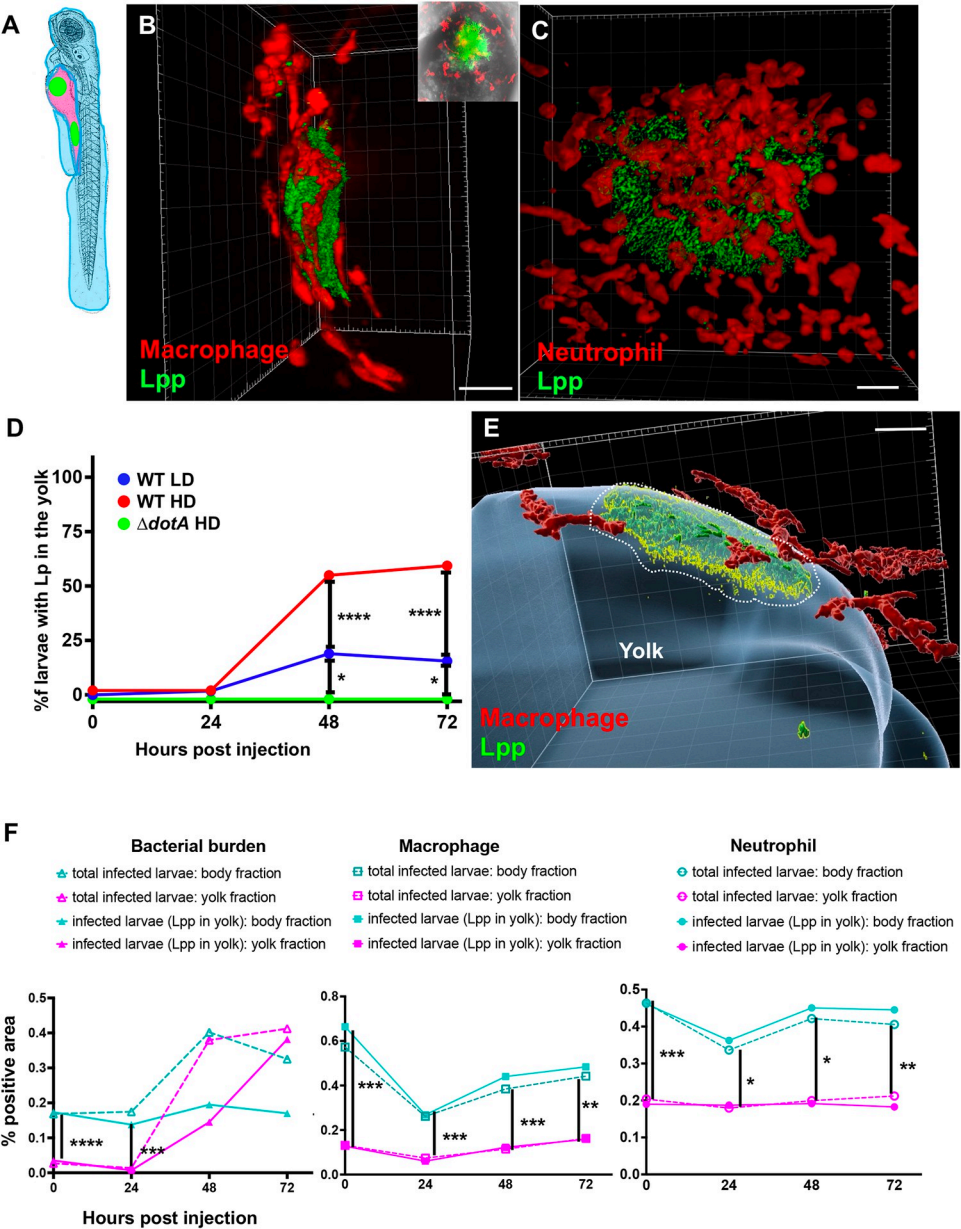
Characterization of the *L*. *pneumophila* foci growing in the yolk region of zebrafish larvae. **A)** Scheme of 72hpf with body (light blue) and yolk region (pink) highlighted; the yolk sustaining *L*. *pneumophila* growing has been indicated with green dots. The scheme of the zebrafish larvae has been adapted from [35] and has been previously modified from [87]. **B**) Imaris 3D reconstruction and volume rendering of the *L*. *pneumophila* growth in the yolk of 72 hpi *mfap4*: mCherry larva (red macrophages) injected in the bloodstream with HD of WT-GFP at 72hpf, shown laterally. Inset shows the maximum intensity projection of the *L*. *pneumophila* foci of the same larva mounted ventrally. Scale bar = 20mm. Related to S3 Movie. **C)** Imaris 3D reconstruction and volume rendering of the *L*. *pneumophila* growth in the yolk of *lyz*:DsRed (red neutrophils) infected larva at 72 hpi, showed laterally. Scale bar = 20mm. Related to S4 Movie. **D)** Quantification of bacterial burden in the yolk over time. Larvae injected with LD, HD WT, or Δ*dotA* HD were imaged over time, and then scored for the GFP + bacteria absolute presence in the yolk for each larva over time. Larvae were scored as “infected” when they showed at least one small detectable GFP+ dot. Data obtained were plotted as % of larvae with bacteria in the in the yolk upon LD (blue curve), HD (red curve) WT or Δ*dotA* HD (green curve) injection over time. 11 independent experiments have been plotted (representing a total of n = 69 WT LD; n = 58 WT HD; n = 54 Δ*dotA* HD infected larvae) **E)** Time frame extracted from a 4D acquisition of *L*. *pneumophila* growth in the yolk between 48 and 72 hpi of *mfap4*: mCherry larva (red macrophages) injected in the bloodstream with HD of WT-GFP shown laterally. Imaris 4D reconstruction and volume rendering of the bacteria aggregate and interaction with macrophages. The yolk has been manually highlighted (in blue) with Imaris. Scale bar: 10. Related to S5 Movie. **F)** Quantification of bacterial burden in the whole body, in the body or in the yolk region versus macrophage or neutrophil quantification in the body or in the yolk region in HD WT-GFP infected larvae followed over time. Two independent experiments plotted for each phagocyte type (total of 11 larvae for macrophage and 11 larvae for neutrophil quantification). Quantification of the fluorescence images (GFP bacteria and RFP leukocytes) was done using CellProfiler software. P < 0.05 was considered statistically significant (symbols: **** P < 0.0001; ***P < 0.001; **P < 0.01; *P < 0.05). No symbol on graphs means that not statistically differences were observed.

To characterise the *L*. *pneumophila* foci identified in the yolk region of zebrafish larvae, we used high resolution fluorescence microscopy of HD of WT-GFP *L*. *pneumophila* injected in the bloodstream of 72hpf zebrafish larvae and analysed them at 72 hpi. This analysis confirmed that these bacterial structures localize in the yolk and or in the yolk tube region (Fig 2D, S1 Movie). *L*. *pneumophila* foci in the yolk region are highly complex aggregate-like structures of long filamentous bacteria. Moreover, upon injection of HD of WT-GFP in transgenic (Tg) zebrafish larvae Tg (*kdrl*::mCherry)^is5^ (blood vessels, fluorescently labelled red) larvae, we showed that the fast growing bacterial aggregates localize close to the blood vessels, mostly below and probably above the yolk cell. Single bacteria near the aggregates localized within the blood vessels, indicating that the bacteria can cross the endothelial barrier to reach the yolk region (Fig 2E, S2 Movie). To analyse macrophage and neutrophil interactions with the bacterial aggregates in the yolk region, we injected HD of WT-GFP in: Tg*(mfap4*::*mCherryF*) (herein referred as *mfap4*:mCherryF) (macrophages, fluorescently labelled red), or Tg(*Lyz*::*DsRed)*^nz50^ (herein referred as *lyz*:DsRed) (neutrophils, fluorescently labelled red). or Tg(*kdrl*::mCherry)^is5^ zebrafish larvae. At 72 hpi macrophages accumulated around the yolk region containing *L*. *pneumophila* but did not seem to be able to engulf the bacterial aggregates (Fig 3A, S3 Movie). Similarly, upon injection of HD of WT-GFP in *lyz*:DsRed larvae, at 72 hpi neutrophils accumulated around the growing bacterial aggregates, but seemed also unable to engulf them (Fig 3C, S4 Movie). Strikingly, quantification analyses showed that bacterial colonisation of the yolk of zebrafish larvae injected with the T4SS deficient Δ*dotA* mutant strain, never took place, suggesting that zebrafish susceptibility to *L*. *pneumophila* infection and yolk penetration depends on a functional T4SS system or the T4SS deficient Δ*dotA* mutant enters a viable but non culturable state (Fig 3D). It should be noted that the yolk is the only food source of the larvae during this developmental stage. The fast proliferation of the bacteria in the yolk region probably depletes its nutritional content, and replicating bacteria may also release toxic compounds, leading to larvae death.

To gain deeper insight into the exact anatomical localisation of the bacteria in the yolk region, we performed high resolution confocal time lapse acquisitions of HD of WT-GFP bloodstream injected larvae harbouring red macrophages between 48 and 72 hpi. We observed that *L*. *pneumophila* foci seem to localize both above but also below the plasmatic yolk membrane, suggesting that they can cross it, and that professional phagocytes remain above the growing bacterial aggregates failing to engulf them (Fig 3E, S5 Movie). Live imaging showed that both macrophages and neutrophils accumulated around bacteria proliferating in the yolk region. To investigate if professional phagocytes were recruited to the bacteria from other sites, or if only the population located on the yolk was involved, we quantified macrophages and neutrophils in the body and in the yolk of the whole larva over time, focusing on HD of WT-GFP infected larvae. We then separated the larvae with bacterial burden in the yolk from the total population of infected larvae, to specifically analyse the recruitment of professional leukocytes to the bacteria growing in the yolk. Following the same criteria as above, we quantified the bacterial burden of the infected larvae over time. This quantitative analysis showed that macrophages and neutrophils that accumulated where the bacteria are seen in the yolk by 48 hpi, were the ones located on the yolk surface, and that no professional phagocyte population was recruited from the other parts of the body (Figs 3F and S6). Moreover, this analysis confirmed that the bacterial burden increased in the yolk while decreasing in the body and it also revealed a transient decrease of professional phagocyte populations at 24h in infected larvae upon HD of WT-GFP injection (Fig 1E).

Thus, blood-borne *L*. *pneumophila* can invade the yolk sac of zebrafish larvae, a previously undescribed phenotype of bacterial infection in this model. Once in the yolk, the bacteria replicate extensively, forming complex, organized, aggregate-like structures that cannot be removed by macrophages and neutrophils, thereby avoiding the host’s immune control and clearance, and eventually causing the death of the larvae.

### Infection of zebrafish larvae with high doses of *L. pneumophila* leads to macrophage and neutrophil death

**Fig 4:**
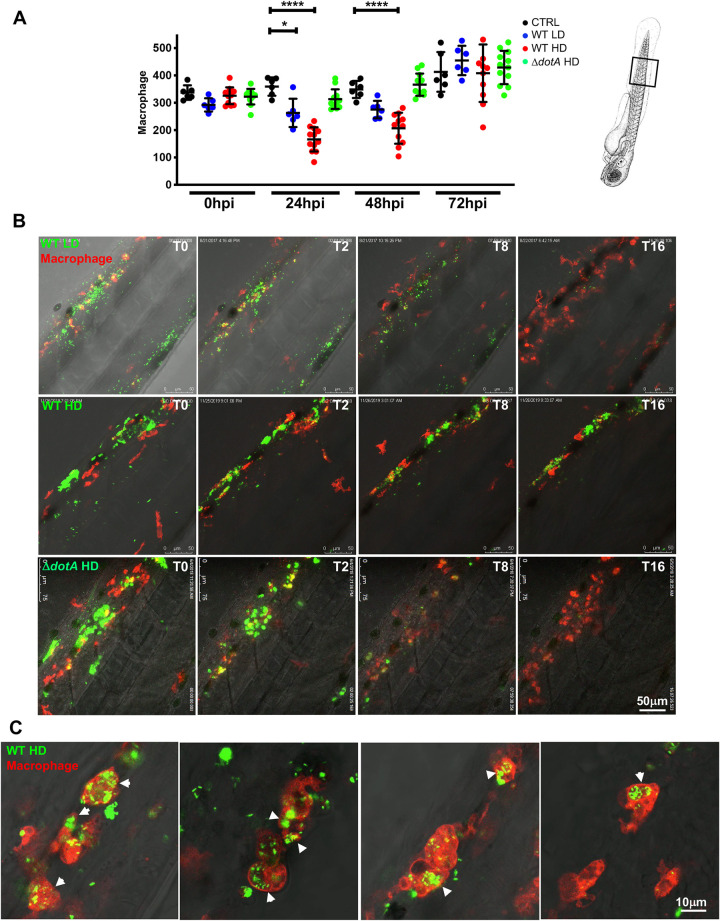
*L*. *pneumophila* high dose injection results in (systemic) macrophage and neutrophil death. **A)** Macrophage counts of control larvae (black symbols) or upon Low Dose (blue symbols) or High Dose of WT-GFP (red symbols), or High Dose (green symbols) of Δ*dotA* -GFP injection. The scheme of the zebrafish larvae has been adapted from [35] and has been previously modified from [87]. Macrophages were counted manually from images taken on live infected larvae over time from T0 to T72 hpi, using ImageJ software, and results were plotted using GraphPad Prism software. Mean±SEM are also shown (horizontal bars). Data plotted are from two pooled independent experiments (n = 12 larvae scored for each condition). P < 0.05 was considered statistically significant (symbols: **** P < 0.0001; ***P < 0.001; **P < 0.01; *P < 0.05). No symbol on graphs means that not statistically differences were observed. **B)** Frames extracted from maximum intensity projection of *in vivo* time-lapse confocal fluorescence microscopy of 72hpf Tg*(mfap4*::*mCherryF*) larvae injected in the bloodstream (iv) with a LD, HD (of WT-GFP or a HD of Δ*dotA*-GFP (upper panel) or Tg(*LysC*::*DsRed)*^*nz50*^ in the bloodstream with a LD, HD of WT-GFP or a HD of Δ*dotA*-GFP (lower panel) to follow macrophage bacteria interaction over time during the first 16 hpi. Overlay of green (*L*. *pneumophila*) and red (leucocytes) fluorescence of the caudal area of the larvae (region boxed in the scheme on the right of the panel) is shown. BF helps for anatomical region indication. Representative of n = 12 to 16 injected larvae for each condition. Scale bar: 50μm. See also related S6 Movie. **C)** macrophage *L pneumophila* interaction at 72 hpi captured at high resolution upon HD WT injection. Bacteria inside zebrafish macrophages suggesting the establishment of a replicative niche (arrows), as documented for cultured mammalian macrophages or amoebae. Representative of n = 15 scored infected larvae. Overlay of green (GFP bacteria), red (mCherry macrophages) and BF is shown. Scale bar = 10mm.

**Fig 5:**
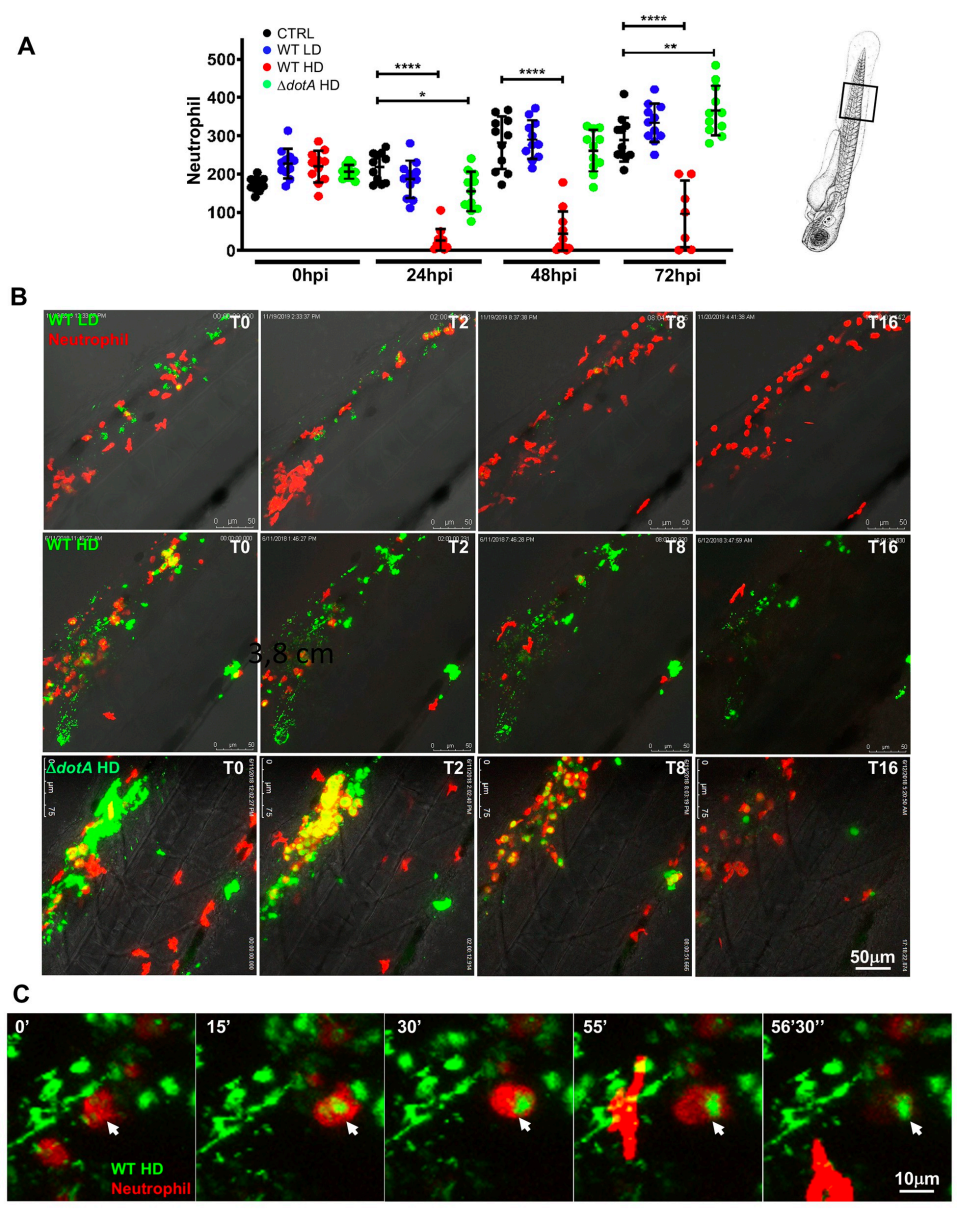
*L*. *pneumophila* interaction with neutrophils and neutrophil counts upon bloodstream injection of LD, HD WT GFP or HD ΔdotA *L. pneumophila*. **A)** Neutrophil counts from control larvae (CTRL, black symbols) or Low Dose or High Dose of WT-GFP (blue or red symbols), or High Dose of Δ*dotA*-GFP (green symbols) injected larvae. The scheme of the zebrafish larvae has been adapted from [35] and has been previously modified from [87]. Data plotted in the same way as for macrophage counts in Fig 4. Two pooled independent experiments (n = 10 larvae scored for each condition). P < 0.05 was considered statistically significant (symbols: **** P < 0.0001; ***P < 0.001; **P < 0.01; *P < 0.05). No symbol on graphs means that not statistically differences were observed. **B)** Frames extracted from maximum intensity projection of *in vivo* time-lapse confocal fluorescence microscopy of 72hpf Tg(*LysC*::*DsRed)*^*nz50*^ in the bloodstream (iv) with a LD, HD of WT-GFP or a HD of Δ*dotA*-GFP (lower panel) to follow neutrophil interaction with *L*. *pneumophila* immediately upon injection for 16 hpi. Images were taken from time lapse at different time points (0 hpi, 2 hpi, 4 hpi, 8 hpi and 16 hpi). Overlay of green (*L*. *pneumophila*) and red (neutrophils) fluorescence of the caudal area of the larvae (region boxed in the scheme on the right of the panel) is shown. Data representative of n = 12 to 16 larvae scored. Scale bar: 50μm. See also related S7 Movie. **C)** Details of a dying phagocytosing neutrophil, progressively rounding-up and loosing fluorescence upon HD WT injection. Arrowheads point to the dying phagocytosing neutrophils. Representatives of n = 14 larvae scored. Scale bar = 10mm. See also related S8 Movie.

In human infection, alveolar macrophages are the primary cell type infected by *L*. *pneumophila* supporting its intracellular replication. Following infection, neutrophils are recruited to the lung and are key players for controlling infection as they possess antimicrobial activity and kill *L*. *pneumophila* [31]. In this study, the recruitment of professional leukocytes to bacteria in the yolk following HD ofWT-GFP larvae infection revealed a decrease of professional phagocyte populations over time (Fig 3F). Thus, to analyse whether zebrafish infection mirrors human infection, we monitored the behaviour of zebrafish macrophages or neutrophils over time. The zebrafish larvae *mfap4*:mCherryF and *lyz*:DsRed were injected with LD or HD of WT-GFP or with high doses HD of Δ*dotA*-GFP. Infected live larvae were monitored using widefield fluorescence microscopy and the number of leukocytes per larva was assessed by counting fluorescent macrophages and neutrophils over time until 72 hpi. We observed that upon injection of HD of WT-GFP, the macrophage count decreased dramatically at 24 hpi and 48 hpi, but started to increase at 72 hpi (Fig 4A). Neutrophil counts gave similar results at 24 and 48 hpi upon injection of HD of WT bacteria, as there was a dramatic decrease observed in neutrophil numbers by 24 hpi. However, the neutrophil counts were still decreased at 72 hpi (Fig 5A). In contrast macrophage and neutrophil counts remained unaffected upon injection of equal amounts of the avirulent Δ*dotA*- GFP strain, with a slight increase of neutrophil numbers at 72 hpi, suggesting that phagocyte death is linked to a functional T4SS system (Figs 4A and 5).

Taken together, these results show that high dose *L*. *pneumophila* infection leads to a decrease in the number of professional phagocytes dependent on the T4SS, like what is seen during human infection by *L*. *pneumophila* or *Mycobacterium tuberculosis* [31,32]

### Macrophages are the primary cells to phagocytise blood-borne *Legionella pneumophila* and neutrophils co-operate to decrease bacterial load

As macrophages and neutrophils are the phagocytes known to interact with *L*. *pneumophila* we analysed phagocyte-*L*. *pneumophila* interactions *in vivo* by injecting *mfap4*:mCherryF or *lyz*:DsRed 72hpf larvae with WT-GFP or Δ*dotA*-GFP and recorded phagocyte-*L*. *pneumophila* interactions using high resolution confocal microscopy. This showed that upon injection of LD WT-GFP, macrophages immediately contacted and engulfed blood-borne bacteria. Macrophages were continuously recruited to the site of injection and by 16 hpi the bacteria were mostly undetectable while macrophage numbers increased (Fig 4B top panel, S6 Movie). Macrophages that had engulfed a large amount of *L*. *pneumophila* stopped moving and rounded-up. Similarly, the inhibition of the migration of phagocytes by *L*. *pneumophila* has been observed previously during infection of RAW 264.7 macrophages and the amoeba *Dictyostelium discoideum* and *Acanthamoeba castellanii*, [33,34]. In contrast, zebrafish injected with HD of WT-GFP were not able to restrict the bacterial growth by 16 hpi. Injection of HD of *L pneumophila* led to the formation of big bacterial aggregates, that were not easily engulfed and cleared by macrophages, as previously shown for bacterial aggregates proliferating in the yolk region (Fig 4B, second panel, S6 Movie). Remarkably, macrophages were very efficient in engulfing and rapidly clearing high doses and big bacterial aggregates of blood-borne Δ*dotA*-GFP bacteria. By 10 hpi most of the Δ*dotA* bacteria and bacterial aggregates had been engulfed and cleared as suggested by the diffuse GFP staining in phagocytes (Fig 4B, bottom panel, S6 Movie). However, upon infection with a HD of WT-GFP, bacteria were not completely cleared and at 72 hpi *L*. *pneumophila* were found in macrophages, suggesting that the bacteria are also replicating in macrophages of zebrafish larvae. Indeed, high resolution confocal microscopy showed that at 72 hpi, *L*. *pneumophila* are also found inside of macrophages in structures resembling replicative vacuoles (Fig 4C).

The analyses of *L*. *pneumophila*-neutrophil interactions showed that these cells engulfed the bacteria trapped in the mesenchyme around the site of injection, but they were less efficient at clearing blood-borne bacteria. This has also been previously observed for infection of zebrafish larvae with *Escherichia coli* or *Shigella flexneri* [26,35]. Indeed, upon injection with a HD of WT-GFP, neutrophils failed to restrict *L*. *pneumophila*, leading to massive death of infected neutrophils, they rounded up and lost their fluorescence (Fig 5B, second panel; S7 Movie; Fig 5C; S8 Movie). In sharp contrast, neutrophils very efficiently engulfed and cleared large amounts of Δ*dotA*-GFP aggregated and trapped in the mesenchyme (Fig 5B, lower panel, S7 Movie) as well as when fish were injected with LD of WT-GFP (Fig 5B upper panel, S7 Movie).

Altogether this shows that upon low dose bloodstream injection of *L*. *pneumophila*, macrophages and neutrophils efficiently cooperate to eliminate most of the injected bacteria within 20–24 hpi, with macrophages playing the primary role. However, *L*. *pneumophila* seems to persist upon high dose WT-GFP injection, and the observation of structures resembling large vacuoles containing *L pneumophila* at 72 hpi suggests that they can replicate in zebrafish macrophages. In contrast, neutrophils interact with *L*. *pneumophila* by quickly engulfing bacteria trapped in the mesenchyme near the site of injection but are less efficient in clearing blood-borne bacteria.

### Macrophages are the first line defence restricting *L. pneumophila* infection

In humans, innate immune responses, based essentially on the activity of professional phagocytes and the induction of pro-inflammatory cytokine genes, are the key players to control and restrict *L*. *pneumophila* proliferation. Hence, human disease develops primarily in immunocompromised individuals [10]. To investigate whether the phagocytes of the innate immune system, macrophages and neutrophils, are also responsible for controlling *L*. *pneumophila* infection in zebrafish larvae, we selectively and transiently depleted macrophages or neutrophils, and infected these “immunocompromised” larvae with *L*. *pneumophila*. Depletion of macrophages was achieved by knocking down the expression of s*pi1b*, a transcription factor involved in early myeloid progenitor formation. A low dose of s*pi1b* morpholino was reported to impact macrophages without affecting neutrophils [36]. We monitored the effect of low doses s*pi1b* morpholino injection on macrophage and neutrophil populations in double transgenic larvae with green neutrophils (*mpx*:GFP) and red macrophages (*mfap4*:mCherryF). The specific depletion of macrophages was confirmed by counting macrophages and neutrophils at 72hpf (S7A Fig).

**Fig 6:**
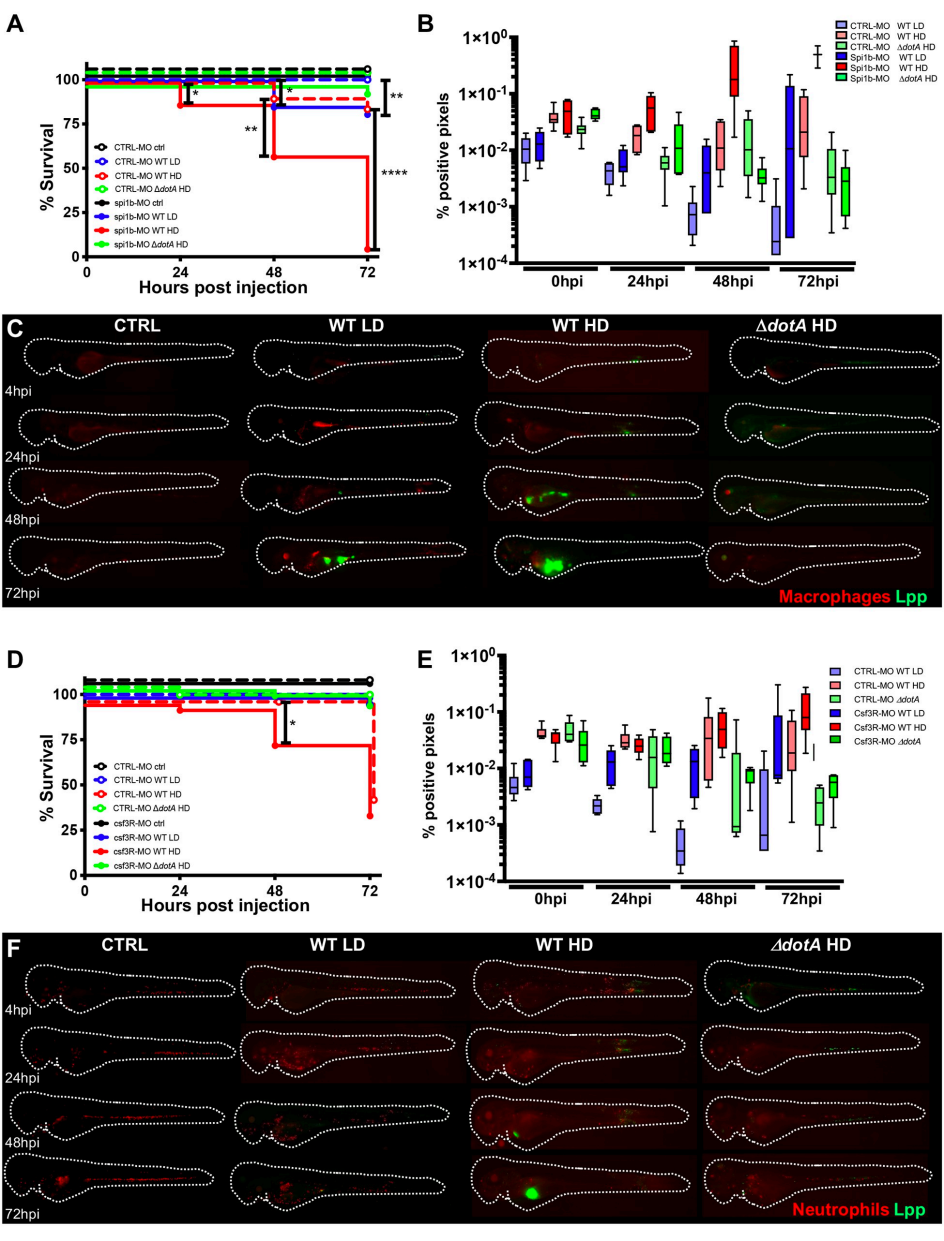
Macrophages are crucial to restrict *Legionella pneumophila* dissemination. **A)** Survival curves of CTRL morphant zebrafish larvae injected with a Low Dose (LD) (blue dashed curve, n = 34 larvae) or a High Dose (HD) (red dashed curve, n = 34) of WT-GFP, or with a HD (green dashed curve, n = 24) of Δ*dotA* -GFP, and *spi1b* morphant zebrafish larvae injected with a LD (blue curve, n = 48) or a HD (red curve, n = 48) of WT-GFP, or with a High Dose (HD) (green curve, n = 48) of Δ*dotA* -GFP. Non-injected CTRL morphant fish (black dashed curve, n = 48), and *spi1b* morphant fish (black curves, n = 48) were used as control. Infected and control larvae were incubated at 28°C. Data plotted are from two pooled independent experiments. **B)** and **E**) Bacterial burden evaluation by quantification of % of fluorescent pixel counts on individual injected larvae followed over time by 0 to 72 hpi. Each larva was imaged daily, and images were analysed with Fiji for bacterial burden evaluation. One experiment per phagocyte type plotted, 6 larvae for each condition. **D)** Survival curves of CTRL morphant zebrafish larvae injected with a LD (blue dashed curve, n = 36) or a HD (red dashed curve, n = 36) of WT-GFP, or with a HD (green dashed curve, n = 24) of Δ*dotA* -GFP, and *csf3r* morphant zebrafish larvae injected with a LD (blue curve, n = 24) or a HD (red curve, n = 36) of WT-GFP, or with a HD (green curve, n = 36) of Δ*dotA* -GFP. Non-injected CTRL morphant fish (black dashed curve, n = 48), and *csf3r* morphant fish (black curve, n = 36) were used as control. Data plotted are from two pooled independent experiments. Significant differences are highlighted with stars (see experimental procedure for statistical analysis) **C)** and **F)** Representative images of *L*. *pneumophila* dissemination, determined by live imaging using a fluorescence stereomicroscope, of Tg*(mfap4*::*mCherryF*) *spi1b* morphant larvae (**C**) and of Tg(*LysC*::*DsRed)*^*nz50*^ (**F)** *csf3r* morphant larvae non infected, or infected with a LD or a HD of WT-GFP, or a HD of Δ*dotA*-GFP. The same infected larvae were live imaged 4h, 24h, 48h, and 72h post *L*. *pneumophila* injection. Overlay of GFP and mCherry fluorescence is shown. P < 0.05 was considered statistically significant (symbols: **** P < 0.0001; ***P < 0.001; **P < 0.01; *P < 0.05). No symbol on graphs means that not statistically differences were observed.

We then infected macrophage depleted larvae (s*pi1b* knockdown) by intravenous injection of LD or HD of WT-GFP. Regardless of the infection dose, a dramatic decrease in larvae survival was observed, as even injection of low doses of WT-GFP resulted in the death of 30% of the larvae (Fig 6A). When injecting HD of WT-GFP nearly all the infected larvae died by 72 hpi, with the earliest deaths starting 48 hpi (Fig 6A). In contrast, s*pi1b* knockdown larvae injected with HD of Δ*dotA*-GFP did not show impaired survival (Fig 6A). The increased mortality correlated with an increased but not significantly different bacterial burden in the s*pi1b* knockdown larvae compared to control larvae (Figs 6B and S2C). Intravital imaging of infected s*pi1b* knock down larvae also showed that both LD and HD of WT-GFP failed to be cleared and that the bacteria established a replicative niche in the yolk, where they proliferated extensively (Fig 6C). This highlights, that macrophages are critical to restrict the onset of infection and *L*. *pneumophila* proliferation *in vivo*. Furthermore, these results also suggest that neutrophils, which are not depleted in s*pi1b* knockdown larvae, fail to control *L*. *pneumophila* infection in the absence of macrophages.

To analyse the role of neutrophils in controlling the infection, neutrophil development was disrupted by knocking down the G-CSF/GCSFR pathway using *csf3R* morpholino, previously reported to decrease up to 70% of the neutrophils present [37–39]. First, we monitored the efficiency of the *csf3R* morpholino knockdown in double transgenic larvae, and confirmed that 75% of the neutrophil population was depleted, while macrophage numbers were only slightly decreased (S7B Fig). When HD of Δ*dotA*-GFP was the bacterial burden remained unchanged, as observed in infections of macrophage-depleted larvae (Fig 6D and 6E). However, when neutrophil-depleted larvae were injected with HD of WT-GFP, larvae survival significantly decreased and bacterial burdens slightly increased by 48 hpi (Figs 6D, 6E and S2D). Intravital imaging revealed that those *csf3R* knockdown larvae that were unable to control *L*. *pneumophila* infection showed bacterial proliferation in the yolk comparable to WT larvae (Fig 6F).

These results show that both macrophages and neutrophils are required for restricting and controlling *L*. *pneumophila* infection in the zebrafish model, but macrophages play the main role. Although neutrophils contributed less to clear the bacteria upon bloodstream injection, neutrophils might impact the infection outcome through cytokine release that can modulate macrophage activity.

### Key pro-inflammatory cytokine genes are induced upon *L. pneumophila* infection of zebrafish larvae

**Fig 7:**
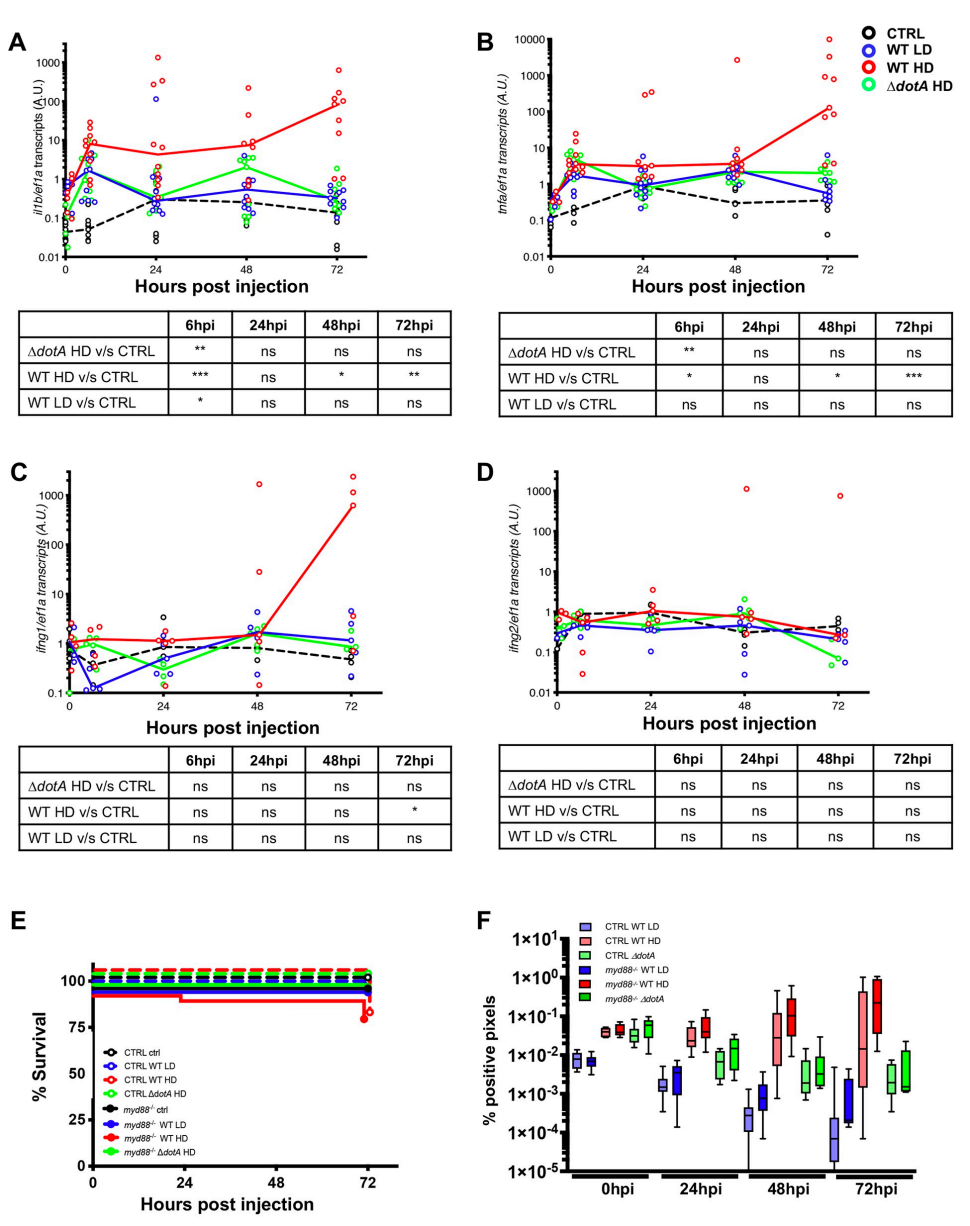
Cytokine gene induction upon *L*. *pneumophila* infection and zebrafish larva Immunity to *L*. *pneumophila* is independent from signalling through MyD88 or compensated by other signalling pathways. **A-D)** Cytokine gene (*il1b*, *tnfa*, *ifng1*, *ifng2*) induction was measured from non-injected larvae as control (CTRL, dashed black curves) and individual zebrafish larvae injected with a LD (blue curves) or a HD (red curves) of WT-GFP, or a HD of Δ*dotA*-GFP (green curves). Data plotted are from 2 pooled experiments (n = 10 larvae for each condition) for *il1b* and *tnfa*, and from 1 experiment (n = 5 larvae for each condition) for *ifng1* and *ifng2*; individual values are shown, and curves correspond to the medians. Statistical analyses are shown in the table under each graph. **E)** Survival curves of CTRL zebrafish larvae injected with WT-GFP Low Dose (LD) (blue dashed curve) or High Dose (HD) (red dashed curve), or with Δ*dotA* -GFP HD (green dashed curve), and *myd88*^*hu3568*^ mutant zebrafish larvae injected with WT-GFP LD (blue curve) or HD (red curve), or with Δ*dotA* -GFP HD (green curve). Non-injected CTRL larvae (black dashed curves), and *myd88*^*hu3568*^ mutant larvae (black curves) were used as control. Infected and control larvae (n = 72 fish for *myd88*^*hu3568*^ mutant conditions and n = 57 fish for CTRL conditions) were incubated at 28°C. Data plotted are from 3 pooled independent experiments. **F)** Bacterial burden evaluation by quantification of % of fluorescent pixel counts on individual injected larvae followed over time from 0 to 72 hpi. Each larva was imaged daily, and images were analysed with Fiji for bacterial burden evaluation. Two pooled experiments, 8 larvae for each condition. P < 0.05 was considered statistically significant (symbols: **** P < 0.0001; ***P < 0.001; **P < 0.01; *P < 0.05). No symbol on graphs means that not statistically differences were observed.

Proinflammatory cytokines produced by infected and bystander cells during *L*. *pneumophila* infection of humans and mice play crucial roles in orchestrating host defences to control infection [40,41]. Infected cells produce IL-1α and IL-1β through a mechanism involving myeloid differentiation factor 88 (MyD88)-dependent translational bypass. In contrast, bystander cells produce: IL-6, TNF-α and IL-12 in an IL-1 receptor (IL-1R) dependant way [40,42]. To determine the pro-inflammatory responses of zebrafish larvae during *L*. *pneumophila* infection, we analysed *il1b*, *tnfa*, and *ifng1/2* (orthologues of mammalian *Ifng*) gene expression levels over time by qRT-PCR on RNA isolated from individual infected larvae. We found that infection of zebrafish larvae with LD or HD of WT-GFP induced a rapid (by 6 hpi) and robust induction of *il1b* and *tnfa* gene expression. In larvae injected with LD of WT-GFP the expression levels of *il1b* and *tnfa* started to decrease by 24 hpi, and gradually became undetectable at 72 hpi. In contrast, larvae injected with HD of WT-GFP, expression of *il1b* and *tnfa* did not decrease over time (Fig 7A and 7B) and a significant induction of *ifng1* was observed at 48 hpi (Fig 7C) but not of *ifng2* (Fig 7D). In parallel, we scored the bacterial burden of the infected larvae before the measurement of pro-inflammatory cytokine gene induction at each time point under the microscope, which consistently showed that larvae with increased *il1b* and *tnfa* induction also had high bacterial burdens in the yolk and were not controlling the infection (S8A FIg). These pro-inflammatory responses were T4SS dependent, as zebrafish larvae infected with HD of Δ*dotA*-GFP did not show significant induction of transcription of *tnfa*, *il1b* and *ing1/2* (Fig 7A-7D).

Collectively, these results reveal that key pro-inflammatory cytokines known to orchestrate the host response during *L*. *pneumophila* infection in humans, are also induced in zebrafish larvae, and that cytokine gene induction is sustained when uncontrolled *L*. *pneumophila* proliferation occurs.

### The immune response of zebrafish larvae to L. pneumophila infection is independent of MyD88 signalling

In innate immunity, MyD88 plays a pivotal role in immune cell activation through Toll-like receptors (TLRs). MyD88-deficient mice are highly susceptible to *L*. *pneumophila* infection [43–46], however this is not the case when human macrophages are depleted of MyD88 [47]. Therefore, we sought to analyse which role MyD88 plays in zebrafish larvae during *L*. *pneumophila* infection. We injected *myd88-/-* and WT larvae with LD or HD of WT-GFP, or with HD of Δ*dotA-*GFP and monitored larvae survival and bacterial burden over time as described in Fig 1. Our results show that susceptibility to infection of *myd88*-/- larvae injected with HD of WT-GFP was comparable to that of WT larvae, as only a very slight, but no significant increase of bacterial burden was observed in *myd88*-/- larvae by 24 hpi. When *myd88*-/- larvae or WT larvae were injected with Δ*dotA-*GFP, infection was not established and the bacterial burden decreased over time, indicating that bacteria were cleared (Figs 7E, 7F and S2E). To determine if pro-inflammatory responses were affected in the absence of MyD88 signalling, we analysed *il1b*, *tnfa* and *ing1/2* gene expression levels over time in WT and *myd88*-/- larvae. Our results showed that *il1b*, *tnfa* and *ing1/2* gene expression levels were comparable in the WT control and *myd88*-/- infected larvae for all tested conditions LD of WT-GFP and HD ofΔ*dotA*-GFP (S8B and S8C Fig).

Taken together, our results suggest that MyD88 signalling is not required for the innate immune response against *L*. *pneumophila* infection in the zebrafish larvae, corroborating what has been observed in human cell infection models. However, we cannot exclude that MyD88 signalling may also be functionally compensated by other immune signalling pathways.

## Discussion

In this study, we developed a zebrafish larva infection model for *L*. *pneumophila* and have analysed host pathogen interactions as well as the host innate immune response to infection. We have found that a successful infection of zebrafish larvae by *L*. *pneumophila* depends on: the infection site, the infection dose, the T4SS Dot/Icm and the innate immune response of the host, in particular on the action of macrophages and neutrophils. Wild type zebrafish larvae are susceptible to infection in a dose dependent manner, as larvae injected in the bloodstream with increasing doses of bacteria, developed an infection with bacterial dissemination and replication, concomitant with host death proportional to the initial injected bacterial load. However, the fact that only about 30% of the larvae displayed this phenotype, indicates that the innate immune defence of the larvae against *L*. *pneumophila* infection is relatively efficient. Thus, the establishment of infection in zebrafish larvae is determined not only by the infection dose but also by the capacity of the host immune system to quickly and efficiently fence off the infection.

Like in the zebrafish infection models for *Staphylococcus aureus* and *Shigella*, only blood borne *L*. *pneumophila* can proliferate and induce mortality in zebrafish larvae [48,49]. Once in the blood circulation, bacteria are engulfed and are eliminated by both macrophages and neutrophils in a dose-dependent manner. However, T4SS competent *L*. *pneumophila* are also able to reach the yolk region, cross the endothelium of the yolk vessels and enter the yolk sac region. Once there, *L*. *pneumophila* gains a significant advantage in the pathogen-host arms race and establishes a replicative niche where it proliferates extensively, forming complex, aggregated bacterial structures, located below and above the yolk cell membrane, that professional phagocytes fail to clear. In the yolk sac region *L*. *pneumophila* is protected from professional phagocytes as they are unable to enter the yolk. Extensive proliferation of the bacteria eventually leads to host death, likely due to exhaustion of the nutrients present in the yolk, which are key in supporting the larvae development. Additionally, the bacteria may also release compounds toxic for the larvae. Interestingly, we have also observed that in few cases (less than 5%) the infected larvae were able to extrude the bacterial aggregates growing in the yolk and survived. This host defence mechanism has also been reported in a caudal fin model of *Mycobacterium marinum* infection, where infected zebrafish larvae extruded the bacteria-containing granuloma and during infection with *Aspergillus fumigatus* [50,51].

To our knowledge, the tropism, and the establishment of a replicative niche in the yolk upon pathogen injection in the bloodstream is a unique feature of *L*. *pneumophila*. Our results have shown that, when macrophages and neutrophils are depleted, blood-borne *L*. *pneumophila* can still reach the yolk cell. It seems that *L*. *pneumophila* can cross the venous endothelium of the yolk and reach the nutrient-rich content of the yolk cell. Interestingly, direct yolk cell injection revealed that only the WT but not the T4SS knockout strain is able to replicate and establish a persistent infection in the yolk, irrespective of the dose injected. This result points towards the involvement of the T4SS system and its secreted effectors in infection and replication but also nutrient uptake in the yolk environment. Yolk sac injection has already been used previously for the analyses of *Candida albicans* infection or the study of streptococcal infections, however in these cases the pathogens disseminated from the yolk sac into the animal [52,53], a phenotype not observed when *L*. *pneumophila* was injected in the yolk sac. The preference for the yolk sac as a replicative niche, was further analysed in embryonated chicken eggs. Again only WT *L*. *pneumophila* was able to replicate in the yolk region, not the T4SSS mutant (S5A–S5C Fig), demonstrating that the T4SS is necessary to establish the yolk as a replicative niche, perhaps *via* its roles in nutrient uptake. *L*. *pneumophila* is known to mainly use amino acids as carbon and energy sources for growth [54] and secreted T4SS effectors have been shown to aid in amino acid uptake [55]. However, fatty acids, glucose and/or glycerol also serve as carbon sources during the later stages of the life cycle of *L*. *pneumophila* [56,57], but effectors connected to the uptake of these nutrients have not yet been identified. The yolk cell is composed of a complex and dynamic mixture of different lipids on which the zebrafish larvae rely for nutrition throughout development in the early larva phase. Cholesterol and phosphatidylcholine are the main constituents until 120hpf. Triacylglycerol, phosphatidylinositol, phosphatidylethanolamine, diacyl-glycerol, cholesteryl esters and sphingomyelins are also present in significant concentrations [58]. *L*. *pneumophila* is known to secrete several effectors with lipolytic activity through its T4SS, which could be important for growth in a lipid rich environment like the yolk [59]. In a first attempt to identify one of these effectors, we analysed the growth of a *L*. *pneumophila* mutant for a gene encoding a sphingosine-1 phosphate lyase (*Lp*Spl) [29]. When compared to the WT strain after direct injection in the zebrafish larvae yolk sac, a small but significant difference in larvae mortality was observed for the Δ*spl* strain, suggesting that *Lp*Spl is one of several effectors that might participate in nutrient acquisition from lipids (S5A Fig). However, further analyses are needed to characterize the involvement of *Lp*Spl in nutrient acquisition during infection of the yolk region and to identify other effectors potentially implicated in this phenotype. Additionally, the T4SS might be implicated in counteracting host defence mechanisms as innate immune factors like immunoglobulins and complement have been described in the yolk of zebrafish embryos [60,61].

Studies of *Legionella* infection in humans, guinea pigs and mouse lungs have shown that *L*. *pneumophila* interacts closely with neutrophils and mononuclear phagocytes [62,63]. Professional phagocytes are the main replication niche for *L*. *pneumophila*, with monocytes and macrophages and in particular alveolar macrophages, representing the main cells for replication in the lungs [64–67]. *In vivo* studies in mice have shown that upon lung infection with *L*. *pneumophila*: neutrophils, cDCs, monocytes, and monocyte-like cells are rapidly recruited to the infection site. Although all these cells seem to engulf the bacteria, *L*. *pneumophila* has only been shown to translocate effectors into neutrophils and alveolar macrophages. In zebrafish, macrophages appear during the first days of development, followed by neutrophils a day later, with both forming an efficient immune system that protects the developing fish [27,68–70]. Therefore, the zebrafish larva offers a unique possibility to interrogate the role of innate immune responses to infection [25]. Indeed, macrophage depleted larvae showed a dramatically increased susceptibility to *L*. *pneumophila* infection, as nearly 100% of larvae inoculated with HD of WT and 30% of larvae inoculated with LD of *L*. *pneumophila* WT died from the infection. Hence, macrophages are the first line of infection control against *L*. *pneumophila* and are essential for restricting and controlling blood-borne infections, similar to what has been observed for *Burkholderia cenocepacia or Staphylococcus aureus* zebrafish infection [71,72]. In contrast, when neutrophils were depleted, the innate immune response was less affected, suggesting that macrophages alone are not able to contain high burdens of *L*. *pneumophila* infection (Fig 6).

Human innate immune signalling relies strongly on activation of Toll-like receptors (TLRs) and respective adaptor molecules, all of which are highly conserved in the zebrafish [73,74]. One of these adaptors is MyD88, known as a central player in interleukin 1 receptor (IL-1R) and TLR signalling in humans and mammalian models [75]. MyD88 signalling is crucial for mice to combat *L*. *pneumophila* infection, as it triggers the early secretion of inflammatory cytokines, neutrophil recruitment, and the host immune response to the infection. Consequently, mice that lack MyD88 are highly susceptible to infection [42–45]. However, in MyD88 depleted human macrophages *L*. *pneumophila* replication is not different to replication in WT cells [76] Given the conservation between the zebrafish and the human immune system, we decided to use the zebrafish model to determine the role of MyD88 signalling. Gene expression analyses in zebrafish confirm that MyD88 has no influence on the control of the inflammatory response, as no statistically significant difference in the transcript levels of *il1b*, *tnfa*, *ifng1 or infg2* was observed, further suggesting that activation of the IL1R and certain TLR pathways are not crucial for *L*. *pneumophila* clearance in zebrafish larvae. Taken together, MyD88 signalling does not play a crucial role or may be redundant in the control of the innate immune response to *L*. *pneumophila* infection of zebrafish larvae, suggesting that zebrafish infection mirrors human infection better than the mouse model.

In the mouse model *L*. *pneumophila*-infected macrophages are not producing cytokines, such as tumour necrosis factor (TNF) and interleukin-12 (IL-12), which are necessary to control infection [40,77]. In contrast, infection of zebrafish larvae with LD of *L*. *pneumophila* WT leads to a short induction of *Il1b* transcript levels at 6 hpi before declining to control levels at later time points, suggesting that a short boost of IL-1β is sufficient to control LD of *L*. *pneumophila*. Furthermore, HD of WT *L*. *pneumophila* induced a rapid (by 6 hpi) and robust induction of *il1b* and *tnfa* gene expression. However, this high and long-term induction of IL-1β is not sufficient to warrant infection control, suggesting that the self-regulation of the immune response may be abrogated leading to a constant activation of IL-1β expression. It is even possible that IL-1β release is beneficial for *L*. *pneumophila* replication, as it was shown in human cells that it may indicate an activation of the metabolic state of the bystander cells. Indeed, as reported IL-1β induces a shift towards more metabolically active cells and increased cellular glucose uptake [78], which could aid *L*. *pneumophila* replication.

In conclusion, the zebrafish infection model for *L*. *pneumophila* mimics the immune response observed during human infection and recalls the essentiality of the T4SS for virulence of this pathogen. The unique advantages of the zebrafish model provide exciting possibilities to further explore *L*. *pneumophila* host interactions and to interrogate at the molecular level the bacterial determinants and host factors involved in the dynamics of bacterial dissemination, the molecular basis of yolk region invasion and the interactions of the bacteria with macrophages and neutrophils.

## Materials and methods

### Ethics statement

Animal experiments were performed according to European Union guidelines for handling of laboratory animals (http://ec.europa.eu/environment/chemicals/lab_animals/home_en.htm) and were approved by the Institut Pasteur Animal Care and Use Committee and the French Ministry of Research (APAFIS#31827). The inoculation of embryonated chicken eggs is a standard procedure in diagnostics for multiplication and antigen production of *Legionella* and is not covered by the national law for animal experiments in France (Décret n° 2013–118 du 1er février 2013).

### Zebrafish care and maintenance

Wild-type AB fish, initially obtained from the Zebrafish International Resource Center (Eugene, OR), Tg(*Lyz*::*DsRed)*^nz50^ [79], Tg*(mfap4*::*mCherryF*) (ump6Tg) [39] Tg(mpx:GFP)^i114^ [80], Tg(kdrl::mCherry)^is5^ [81] and *myd88*^*hu3568*^ mutant line (obtained from the Hubrecht Laboratory and the Sanger Institute Zebrafish Mutation Resource) [82], were raised in our facility. Eggs were obtained by natural spawning, bleached according to standard protocols, and kept in Petri dishes containing Volvic source water and, from 24 hours post fertilization (hpf) onwards 0.003% 1-phenyl-2-thiourea (PTU) (Sigma-Aldrich) was added to prevent pigmentation. Embryos were reared at 28°C or 24°C according to the desired speed of development; infected larvae were kept at 28°C. Timings in the text refer to the developmental stage at the reference temperature of 28.5°C. Larvae were anesthetized with 200μg/ml tricaine (Sigma-Aldrich) during the injection procedure as well as during *in vivo* imaging and processing for bacterial burden evaluation or cytokine expression studies.

### Bacterial strains and growth conditions

*Legionella pneumophila* strain Paris carrying the pNT28 plasmid encoding for green fluorescent protein (constitutive GFP) [83], wild-type (WT-GFP) or Δ*dotA*-GFP were plated from -80°C glycerol stocks on N-(2-acetamido)-2-aminoethanesulfonic acid (ACES)-buffered charcoal yeast-extract (BCYE) medium supplemented with 10 μg/ml of chloramphenicol and cultured for 3 days at 37°C. Suspensions were prepared by resuspending bacteria in sterile 1x Phosphate Buffered Saline (PBS) and adjusting the OD 600 according to the desired bacterial concentrations for injection.

### Morpholino injections

Morpholino antisense oligonucleotides (Gene Tools LLC, Philomath, OR, USA) were injected at the one to two cell stage as described [84]. A low dose (4ng) of *spi1b* (previously named pu1) translation blocking morpholino (GATATACTGATACTCCATTGGTGGT) [85] blocks macrophage development only but can also block neutrophil development when it is injected at a higher dose (20ng in 2nl). The csf3r translation blocking morpholino (GAACTGGCGGATCTGTAAAGACAAA) (4ng) [37] was injected to block neutrophil development. Control morphants were injected with 4ng control morpholino, with no known target (GAAAGCATGGCATCTGGATCATCGA).

### Amoebae infections

*Acanthamoebae castellanii* was infected with *L*. *pneumophila* wild type expressing GFP and then used to assess if zebrafish develop an infection when ingesting infected amoebae. *A*. *castellanii* was seeded in a flask with infection buffer (4 mM MgSO_4_, 0.4 M CaCl_2_, 0.1% sodium citrate dihydrate, 0.05 mM Fe(NH_4_)_2_(SO_4_)_2_×6H_2_O, 2.5 mM NaH_2_PO_3_, 2.5 mM K_2_HPO_3_), and after 1 hour of attachment the cells were infected with the bacteria at MOI 1. After one hour of infection, the amoebae were washed three times with PBS and fresh infection buffer was added. After 48 hours of infection, the amoebae were carefully washed, detached, and used for the bath immersion experiment.

### Bath immersion

Experiments were performed using the AB or Tg(mfap4::mCherryF) zebrafish lines maintained at 28°C under standard conditions in Volvic water. Bath immersion infections were done with 120hpf larvae that already had a developed swim bladder. Groups of 10 larvae were distributed into 6-well plates containing 4.0 ml/well of Volvic spring water and either 1ml of bacterial suspension, 1ml of *L*. *pneumophila*-containing amoebae, or 1ml of non-infected amoebae, all in PBS. The plates were incubated at 28°C for 24 hours, and then larvae were individually distributed in an individual well in 24 wells culture plates and monitored by imaging using a fluorescence stereomicroscope.

### Zebrafish injection

The volume of injected suspension was deduced from the diameter of the drop obtained after mock microinjection, as described in [84]. Bacteria were recovered by centrifugation, washed, resuspended at the desired concentration in PBS. 72h post-fertilization (hpf) anesthetized zebrafish larvae were microinjected iv or in closed cavities, or the yolk with 0.5-1nl of bacterial suspension at the desired dose (~10^3^ bacteria/nl for Low Dose (LD) and ~10^4^ bacteria/nl for High Dose (HD) as described [26,35]. Infected larvae were transferred into individual wells (containing 1ml of Volvic water + 0.003% PTU in 24-well culture plates), incubated at 28°C and regularly observed under a stereomicroscope, twice a day over time up to 72 hpi.

### Evaluation of the bacterial burden in infected larvae

The bacterial burden was measured routinely by counting the total number of fluorescent pixels corresponding to the GFP channel using the Metavue software 7.5.6.0. Briefly, images corresponding to the GFP channel were adjusted to a fixed threshold that allowed to abrogate the background of the autofluorescence of the yolk. The same threshold was used for all images. The histogram in the “Analyze” menu was used to obtain the number of black and white pixels. As shown in S1A Fig, the percentage of white pixels in each image corresponding to *L*. *pneumophila* was plotted using GraphPad Prism software. This method was chosen to routinely quantify bacterial burden as it allows to follow each infected larva over time (individual larvae were imaged with the fluorescence stereomicroscope daily using the same settings from 0 to 72 hpi).

### Bacterial burden analyses by FACS

Injected zebrafish larvae were collected at 0, 24, 48 and 72 hpi and lysed. Each larva was placed in a 1.5 ml Eppendorf tube and anesthetized with tricaine (200μg/ml), washed with 1ml of sterile water and placed in 150 μl of sterile water. Larvae were then homogenized using a pestle motor mixer (Argos). Each sample was transferred to an individual well of a 96 well plate, counted on a MACSQuant VYB FACS (Miltenyi Biotec) and data analysed using FlowJo version 7.6.5. as shown in S1B Fig. For CFU enumeration, serial dilutions were plated on BCYE agar plates supplemented with10ug/ml chloramphenicol and the *Legionella* Selective Supplement GVPN (Sigma) added according to the manufacturer’s instructions. Plates were incubated for 4–5 days at 37°C and colonies with the appropriate morphology and colour were scored using the G-Box imaging system (Syngene) and enumerated using the Gene Tools software (Syngene) as shown in S1C Fig. Manual quantification was also performed to identify absolute absence or presence of bacteria in the different zebrafish compartments. Larvae with a single GFP dot (indicating the presence of few bacteria) were considered as infected. The resulting statistical presence map was used to follow the evolution of the infection and dissemination of *L*. *pneumophila* in zebrafish larvae over time (Fig 1E).

### Inoculation and quantification of *L*. *pneumophila* strains in *in ovo* experiments

Fertilized chicken eggs purchased from a local producer (Saint-Maurice-sur-Dargoire, Rhône, France) were incubated at 35°C in an egg incubator (Maino, Italy) to maintain normal embryonic development. Eggs were pathogen and antibiotic free. On day 0, 53 embryonated chicken eggs (ECE) were inoculated at 8 days of embryonation (DOE) with either *L*. *pneumophila* WT (n = 19 ECE in total corresponding to 9, 4, 3 and 3 tested in the experiments No.1, 2, 3 and 4 respectively), *L*. *pneumophila ΔdotA* (n = 17 ECE in total corresponding to 8, 4, 2 and 3 tested in the experiments No.1, 2, 3 and 4 respectively) or sterile PBS as control (n = 17 ECE in total corresponding to 7, 4, 3 and 3 tested in the experiments No.1, 2, 3 and 4 respectively). Two-day cultures of Lpp-WT and Lpp-Δ*dotA* on BCYE at 36°C were suspended in PBS at a DO = 2.5 McFarland and 0.5 mL of suspensions or PBS (negative control) were inoculated in the yolk sac of ECE. *L*. *pneumophila* concentration in WT and Δ*dotA* suspensions before ECE injection was quantified at 9.0 log_10_ CFU/mL for each suspension, and considering the injected volume of 0.5 mL, the median amount of *L*. *pneumophila* in the yolk sac of ECE directly after injection was 8.7 log_10_ CFU. After inoculation, ECE were candled every 24 hours to assess embryo viability until day-6 post infection. Embryo that died the day of inoculation (D0) (n = 1, corresponding to one WT-infected and one *ΔdotA*-infected embryo) was discarded for *L*. *pneumophila* quantification as death was probably due to bad inoculation. Dead embryos were stored at 4°C overnight prior to harvesting the yolk sacs. Remaining live embryos at 6-days post injection were euthanized by overnight refrigeration and the yolk sacs were collected. After measuring their volume, yolk sacs were homogenized using gentleMACS Octo Dissociator (Miltenyi Biotec, Germany) and 100 μL of serial dilutions at 10^−2^, 10^−4^ and 10^−6^ were automatically plated using easySpiral automatic plater (Interscience, France) in triplicates on BCYE agar. *L*. *pneumophila* were quantified after 5 days-incubation using Scan 1200 Automatic HD colony counter (Interscience, France). Comparison of survival curves was performed using Log-rank (Mantel-Cox) test. P < 0.05 was considered statistically significant. *L*. *pneumophila* amounts in the yolk sac after death of ECE were estimated, considering both the measured UFC counts in yolk sac and the yolk sac volumes (median (interquartile range) [IQR] volume, 30 [28.7–31.2] mL). Comparison of *L*. *pneumophila* quantifications between WT- and Δ*dotA*-infected embryos was done using Mann-Whitney test. P < 0.05 was considered statistically significant (symbols: **** P < 0.0001; ***P < 0.001; **P < 0.01; *P < 0.05).

### Live imaging, image processing and analysis

Quantification of total neutrophils and/or macrophages on living transgenic reporter larvae upon infection was performed as previously described [35]. Briefly, bright field, DsRed and GFP images of whole living anesthetized larvae were taken using a Leica Macrofluo Z16 APOA (zoom 16:1) equipped with a Leica PlanApo 2.0X lens, and a Photometrics CoolSNAP *HQ2* camera. Images were captured using Metavue software 7.5.6.0 (MDS Analytical Technologies). Then larvae were washed and transferred in a new 24 wells plate filled with 1ml of fresh Volvic water per well, incubated at 28°C and imaged again under the same conditions the day after. Pictures were analysed, and Tg(*lyzC*::*DsRed*) neutrophils or Tg(*mfap4*::*mCherryF*) macrophages manually counted using the ImageJ software (V 1.52a). Counts shown in figures are numbers of cells per image. The quantification of fluorescence images was also done using CellProfiler Software (Broad Institute) using two semi-automatic batch pipelines. Both pipelines normalize the intensity, operate image pre-processing, and use thresholding to calculate the percentage of area positive for macrophage/neutrophils and bacteria, normalized on the whole image area. Both pipelines also take advantage of manual editing to increase identification accuracy and define the yolk area. The positive signal is then automatically masked to calculate the amount of signal in the body or yolk of each zebrafish for all the experiments and produce a.csv file used for subsequent statistical treatment (Figs 3F and S6). High resolution confocal live imaging of injected larvae was performed as previously described [86]. Briefly, injected larvae were positioned in lateral or ventral position in 35 mm glass-bottom-Dishes (Ibidi Cat#: 81158) or in glass bottom- 8well-slides (Ibidi Cat#: 80827). Larvae were immobilized using a 1% low-melting-point agarose (Promega; Cat#: V2111) solution and covered with Volvic water containing tricaine. A Leica SP8 confocal microscope equipped with two PMT and Hybrid detector, a 10X dry (PL Fluotar 10X dry:0.30), 20X IMM (HC PL APO CS2 20X/0.75), or a 40x water IMM (HC PL APO CS2 40X/1.10) objective, a X–Y motorized stage and with LAS-X software, was used to live image injected larvae. To generate images of the whole larvae, a mosaic of confocal z-stack of images was taken with the 10X or 20X objective using the Tile Scan tool of the LAS-X software and was stitched together using the Mosaic Merge tool of the LAS-X software. All samples were acquired using the same settings, allowing comparisons of independent experiments. The acquisitions were post processed with the Lightning tool of the LAS-X software to eliminate noise (deconvolution). After acquisition, larvae were washed and transferred in a new 24-well plate filled with 1 ml of fresh Volvic water per well, incubated at 28°C and imaged again under the same conditions over time. A Leica SPE inverted confocal microscope and a 40x oil IMM objective (ACS APO 40 × 1.15 UV) was also used to live image larvae infected with *L*. *pneumophila* Δ*dotA-*GFP (Figs 4 and 5). The 3D or 4D files generated by the time-lapse acquisitions were processed, cropped, analysed, and annotated using the LAS-X and LAS-AF Leica software. Acquired Z-stacks were projected using maximum intensity projection and exported as AVI files. Frames were captured from the AVI files and handled with Miocrosoft PowerPoint (Microsoft Office 365) software to mount figures. AVI files were cropped and annotated using ImageJ software. Files generated with the LAS-X software were also processed and analysed with the Imaris software version9.5 (Bitplane, OXFORD Instruments) for 3D or 4D reconstruction, surfacing and volume rendering. For the 4D reconstruction of the yolk membrane, we used two factors to draw its position: 1) the shape of the bacteria, that forms a “ceiling “with a net edge 2) the Transmitted Light (bright field) to delimitate the yolk cell, and the putative localization of the yolk membrane. The location of the yolk cell membrane is thus assumed by these parameters and has been manually annotated (S5 Movie) but cannot be formally established.

### qRT-PCR to measure gene expression of cytokine encoding genes

RNA was extracted from individual larvae using the RNeasy Mini Kit (Qiagen). cDNA was obtained using M-MLV H- reverse-transcriptase (Promega) with a dT17 primer. Quantitative PCR was performed on an ABI7300 thermocycler (Applied Biosystems) using Takyon ROX SYBR 2X MasterMix (Eurogentec) in a final volume of 10 μl. Primers used: *ef1a (housekeeping gene used for normalization)*: GCTGATCGTTGGAGTCAACA and ACAGACTTGACCTCAGTGGT*; il1b*: GAGACAGACGGTGCTGTTTA and GTAAGACGGCACTGAATCCA*; tnfa*: TTCACGCTCCATAAGACCCA and CAGAGTTGTATCCACCTGTTA*; ifng-1-1*: ACCAGCTGAATTCTAAGCCAA and TTTTCGCCTTGACTGAGTGAA; *ifng-2*: GAATCTTGAGGAAAGTGAGCA and TCGTTTTCCTTGATCGCCCA.

### Statistical analysis

Normal distributions were analysed with the Kolmogorov–Smirnov and the Shapiro–Wilk tests. To evaluate difference between means of normally distributed data (for neutrophil and macrophage numbers), an analysis of variance followed by Bonferroni’s multiple comparison tests was used. For bacterial burdens (CFU/FACS counts), values were Log10 transformed. For cytokine expression and bacterial burdens (evaluated by fluorescent pixel count, or FACS) non-Gaussian data were analysed with the Kruskal–Wallis test followed by Dunn’s multiple comparison test. P < 0.05 was considered statistically significant (symbols: **** P < 0.0001; ***P < 0.001; **P < 0.01; *P < 0.05). No symbol on graphs means that not statistically differences were observed. Survival data were plotted using the Kaplan–Meier estimator and log-rank (Mantel–Cox) tests were performed to assess differences between groups. Statistical analyses were performed using GraphPad Prism software. Statistical analyses for *in ovo* experiments, were performed using GraphPrism version 9.4.

## Supporting information

S1 Fig Comparison of three methods to estimate the bacterial burden of infected zebrafish larvae at different points post infection. A) For bacterial burden measure by fluorescent pixel counts, the pictures corresponding to the GFP channel were analysed to quantify the percentage of fluorescent pixels using the ImageJ software. Individual larvae injected with WT-GFP Low Dose (LD) (blue symbols) or High Dose (HD) (red symbols) or infected with Δ*dotA*-GFP HD (green symbols) have been plotted and represented as box plot. Two independent experiments pooled, n = 10 larvae per condition). **B**) For FACS analyses, infected larvae were lysed and then GFP bacteria were counted on a MACSQuant VYB FACS (Miltenyi Biotec). One experiment plotted, n = 5 larvae per condition. **C**) CFUs were enumerated by plating serial dilutions of lysed infected larvae in BCYE agar supplemented with Chloramphenicol and *Legionella* Selective Supplement GVPN (Sigma). One experiment plotted, n = 5 larvae per condition. P < 0.05 was considered statistically significant (symbols: **** P < 0.0001; ***P < 0.001; **P < 0.01; *P < 0.05). No symbol on graphs means that not statistically differences were observed.
(TIF)

S2 Fig Bacterial burden evaluation by FACS overtime on individual lysed larvae after *Legionella pneumophila* infection. For FACS analyses, individual infected larvae were lysed and then GFP bacteria were counted on a MACSQuant VYB FACS (Miltenyi Biotec). **A)** Related to Fig 1C: 6 pooled experiments, n = 26 larvae for WT HD (28 for 72h), n = 26 larvae for WT LD (30 for 72h), n = 25 larvae for *ΔdotA* HD (26 for 72h) **B)** Related to Fig 2B: Fluorescent pixel count evaluation overtime upon yolk cell injection. One experiment is plotted. N = 6 larvae for WT HD, n = 6 larvae for WT LD, n = 5 larvae for *ΔdotA* HD**. C)** Related to Fig 6B: 2 pooled experiments, n = 8 Spi1b-MO larvae for WT HD, n = 8 Spi1b-MO larvae for WT LD, n = 5 Spi1b-MO larvae for *ΔdotA* HD, n = 5 control larvae for WT HD, n = 5 control larvae for WT LD and n = 5 control larvae for *ΔdotA* HD. **D)** Related to Fig 6E: 2 pooled experiments, n = 8 Csf3R-MO larvae for WT HD (10 for 72h), n = 8 Csf3R-MO larvae for WT LD (9 for 72h), n = 5 Csf3R-MO larvae for *ΔdotA* HD (4 for 0h), n = 6 control larvae for WT HD (8 for 72h), n = 6 control larvae for WT LD (9 for 72h) and n = 5 control larvae for *ΔdotA* HD (6 for 72h). **E)** Related to Fig 7B: 3 pooled experiments, n = 13 *myd88* larvae for WT HD, n = 13 *myd88* larvae for WT LD (12 for 0h), n = 13 *myd88* larvae for *ΔdotA* HD, n = 10 control larvae for WT HD, n = 10 control larvae for WT LD, n = 10 control larvae for *ΔdotA* HD. P < 0.05 was considered statistically significant (symbols: **** P < 0.0001; ***P < 0.001; **P < 0.01; *P < 0.05). No symbol on graphs means that not statistically differences were observed.
(TIF)

S3 Fig *L*. *pneumophila* invades the yolk only upon bloodstream inoculation and only blood borne *L*. *pneumophila* WT proliferate in the yolk region of zebrafish larvae. **A)** Scheme of 72hpf larva indicating the sites of bacterial injection. The scheme of the zebrafish larvae has been adapted from [35] and has been previously modified from [87]. Site of injection are indicated by green dashed boxes. OV: otic vesicle; HBV: hind brain ventricle; IV: intravenous injection. **B.** Survival curves. 2 experiments pooled; n = 36 larvae for CTRL and OV, 31 for IV, and 33 for HBV injection. **C**) bacterial burden evaluated over time by fluorescent pixel counts. 1 experiment, 6 larvae per condition. **D.** Representative images of *L*. *pneumophila* dissemination, determined by live imaging using a fluorescence stereomicroscope, of zebrafish larvae infected with a HD WT-, in closed compartments (OV, HBV) or in the bloodstream (IV). Infected larvae were live imaged 4h, 24h, 48h, and 72h post *L*. *pneumophila* injection. Only GFP fluorescence is shown. Green autofluorescence of the lens eye (e) or of the gastrointestinal tract (g) is indicated on CTRL larvae. P < 0.05 was considered statistically significant (symbols: **** P < 0.0001; ***P < 0.001; **P < 0.01; *P < 0.05). No symbol on graphs means that not statistically differences were observed.
(TIF)

S4 Fig Bath immersion of 120 hpf zebrafish larvae using *WT L pneumophila* infected *A castellanii*. **A)** Survival curves. **B)** % of larvae with GFP bacteria. A and B: 1 experiment, 30 larvae for WT Lpp-amoebae, 10 for WT Lpp and 10 for amoebae. **C)** Representative fluorescent imaging of larvae with GFP bacteria in the intestinal tract followed over time. The intestinal tractus is highlighted with white dotted lines. Arrowhead points to GFP bacteria being eliminated with the fecal content. D) representative closeup of GFP bacteria in the intestinal tract. P < 0.05 was considered statistically significant (symbols: **** P < 0.0001; ***P < 0.001; **P < 0.01; *P < 0.05). No symbol on graphs means that not statistically differences were observed.
(TIF)

S5 Fig Δ*spl* mutant injected in the yolk and *L*. *pneumophila* WT but not the T4SS mutant proliferates in the yolk of zebrafish and the yolk of chicken eggs upon direct injection. **A).** 72hpf larva: the yolk cell is highlighted in blue and the yolk content in pink. The scheme of the zebrafish larvae has been adapted from [35] and has been previously modified from [87]. **B).** Survival curves of 72hpf larvae upon injection in the yolk of HD WT (red curve), Δ*dot*A (green curve) or Δ*spi* mutant (violet curve) *L*. *pneumophila* strain. CTRL larvae (black curve). One experiment, 24 larvae for each condition. Significant differences are indicated with stars. C) Survival curves of embryonated chicken eggs (ECE) inoculated with WT strain (red, n = 19 ECE in total corresponding to 9, 4, 3 and 3 ECE tested in the experiments No. 1, 2, 3 and 4 respectively), Δ*dot*A strain (green, n = 17 ECE in total corresponding to 8, 4, 2 and 3 ECE tested in the experiments No.1, 2, 3 and 4 respectively) or PBS (black, n = 17 ECE in total corresponding to 7, 4, 3 and 3 ECE tested in the experiments No.1, 2, 3 and 4 respectively). Survival is expressed in percentage and time in days. Comparison of survival curves was performed using Logrank (Mantel-Cox) test. P < 0.05 was considered statistically significant. D, E) Quantification of *L*. *pneumophila* (expressed in log10 CFU) in the yolk sac of WT-infected embryos (n = 19 in total) and Δ*dotA* infected embryos (n = 17 in total), according to the day of mortality of the embryos (D1, D2, D3 and alive at D6 (euthanized) (D) or the experiments (n = 4) (E). The inoculum after infection was estimated by considering the *L*. *pneumophila* count in the inoculum (WT and Δ*dotA*) before injection and the volume of the yolk sac. Comparison of the quantifications of *L*. *pneumophila* WT- or Δ*dotA*-infected embryos was done using the Mann-Whitney test. Medians and interquartile range are represented. P < 0.05 was considered statistically significant (**** P < 0.0001; ***P < 0.001; **P < 0.01; *P < 0.05).
(TIF)

S6 Fig Quantification of bacterial burden in the whole body, in the body or in the yolk region versus macrophage or neutrophil quantification in the body or in the yolk region in HD WT-GFP infected larvae followed overtime. Two independent experiments plotted for each phagocyte type (total of 11 larvae for macrophage or 11 larvae for neutrophil quantification). Quantification of the fluorescent images (GFP bacteria and RFP leukocytes) was done using CellProfiler software (see Material and Methods for details about the pipeline). Bacterial burden quantification was done over the whole larva (red dot) or discriminating the body (light blue dot) form the yolk region (pink dot). Scheme of 72hpf with body (light blue) and yolk region (pink) highlighted; The scheme of the zebrafish larvae has been adapted from [35] and has been previously modified from [87]. The yolk sustaining *L*. *pneumophila* growing has been indicated with green dots. Quantification of macrophage or neutrophil located in body (light blue dot) or yolk (pink dot) over time. P < 0.05 was considered statistically significant (symbols: **** P < 0.0001; ***P < 0.001; **P < 0.01; *P < 0.05). No symbol on graphs means that not statistically differences were observed.
(TIF)

S7 Fig Macrophage and neutrophil depletion by morpholino: evaluation of the impact on the non-depleted leukocyte population. Comparison of the impact of *spi1b* morpholino injection that blocks macrophage development or csf3r morpholino injection that blocks neutrophil development were administered. Macrophages (red symbols) and neutrophils (green symbols) were counted in CTRL (open symbols) or morphant (full symbols) conditions. **A)** effect of spe1b morpholino on macrophages and neutrophils, showing that spe1b morpholino injection leads to the specific depletion of macrophages and not neutrophils. Related to Fig 4: 2 plotted experiments, n = 10 larvae per group. B) effect of Csf3R morpholino on macrophages and neutrophils, showing that Csf3R morpholino injection leads to the specific depletion neutrophils and slightly impairs the number of macrophages. Related to Fig 5: 2 plotted experiments, n = 10 larvae per group.
(TIF)

S8 Fig **A. Correlation between bacterial burden (evaluated by fluorescence on individual injected larvae before RNA extraction) and cytokine gene induction at 48 and 72 hpi upon bloodstream injection LD, HD WT or HD Δ*dot*A *L pneumophila* strain.** Control non injected, HD WT or HD Δ*dot*A injected larvae were scored under the fluorescent microscope for evaluating bacterial burden immediately before to be lysed and processed for RNA extraction. “-“, “+” to “+++ “respectively indicate no or in, creasing bacterial burden. “-”and “+” symbols were also used to respectively indicate infected dead or live larvae. Related to Fig 7A-7D. **C-D) Cytokine gene (*il1b*, *tnfa*, *ifng1/2*) induction is independent from Myd88 signalling in *L pneumophila* HD WT infected zebrafish larvae**.Cytokine gene induction was measured from individual *myd88*^*hu3568*^ mutant larvae injected with a HD (red curves) of WT-GFP and non-injected fish (CTRL, black curves). The same colours are used for individual CTRL non injected (black dashed) or HD WT injected (red dashed) zebrafish curves. Data plotted are from one experiment (n = 5 larvae for each condition); individual values are shown, and curves correspond to the medians. There is no statistically significant difference between CTRL and *myd88*^*hu3568*^ mutant curves over time for all the conditions analysed. Related to Fig 7E and 7F.
(TIF)

S1 Movie *L*. *pneumophila* growing in the yolk region at 72 hpi: localization in the yolk in AB wild type larva. AB wild type larva 72hpf was injected in the bloodstream with HD of *L*. *pneumophila* WT-GFP, and was analyzed using confocal high microscopy at 72 hpi, to study the behavior of the highly growing bacteria in the yolk region. The infected larva was mounted laterally and acquired using a 20X oil-immersion objective. The acquired Z-stack was deconvolved using Leica Lightening Plug-in and processed for 3D visualization and volume rendering, using IMARIS 9.6 (Bitplane). Note the complex, filamentous, highly aggregate structures (green) formed by the growing *Legionella* in the yolk region (visualized by the bright field).
(MP4)

S2 Movie *L*. *pneumophila* growing in the yolk region at 72 hpi: interactions with blood vessels. *kdrl*:mCherry (red blood vessels) 72hpf larva was injected in the bloodstream with HD of *L*. *pneumophila* WT-GFP, and was analyzed with confocal high microscopy at 72 hpi, to study the behavior of the highly growing bacteria in the yolk region and their interactions with the yolk vasculature. The infected larva was mounted laterally and acquired using a 20X oil-immersion objective. The acquired Z-stack was deconvolved using Leica Lightening Plug-in and processed for 3D visualization and volume rendering, using IMARIS 9.6 (Bitplane). The interactions of the blood vessels (red cells) with the growing bacterial aggregates (green), and the yolk region (bright field) are shown at various magnifications (scale bar indicated on the movie) and various rotations angles to highlight the complex filamentous bacterial structures and their interactions with the blood vessels. Due to the peculiar yolk composition and thickness, it was impossible to acquire the fluorescence of the bacteria growing inside the yolk region distal to the objective, thus appearing as big dark spots.
(MP4)

S3 Movie *L*. *pneumophila* growing in the yolk region at 72 hpi: interactions with macrophages. *Mfap4*: mCherry (red macrophages) 72hpf larva was injected in the bloodstream with HD of *L*. *pneumophila* WT-GFP, and was analyzed with confocal high microscopy at 72 hpi, to study the behavior of the bacteria growing in the yolk region and their interactions with macrophages. The infected larva was mounted ventrally and acquired using a 40X water-immersion objective. Only the yolk region containing the bacterial aggregates was imaged. The acquired Z-stack was deconvolved using Leica Lightening Plug-in and processed for 3D visualization and volume rendering, using IMARIS 9.6 (Bitplane). The interactions of macrophages (red cells) with the growing bacterial aggregates (green), and the yolk region (bright field) are shown at various magnifications (scale bar indicated on the movie) and various rotation angles to highlight the complex filamentous bacterial structures and the recruited macrophages, that recognize the growing bacteria, but fail to penetrate the yolk content, and to engulf the bacterial aggregates. Due to the peculiar yolk composition and thickness, it was impossible to acquire the fluorescence of the bacteria growing inside the yolk distal to the objective, thus appearing as big dark spots.
(MP4)

S4 Movie *L*. *pneumophila* growing in the yolk region at 72 hpi: interactions with neutrophils. *Lys*:DsRed (red neutrophils) 72hpf larva was injected in the bloodstream with HD of *L*. *pneumophila* WT-GFP, and was analyzed with confocal high microscopy at 72 hpi, to study the behavior of the bacteria growing in the yolk region and their interactions with neutrophils. The infected larva was mounted laterally and acquired using a 20X oil-immersion objective. The acquired Z-stack was deconvolved using Leica Lightening Plug-in and processed for 3D visualization and volume rendering, using IMARIS 9.6 (Bitplane). The interactions of neutrophils (red cells) with the growing bacterial aggregates (green), and the yolk region (bright field) are shown at various magnifications (scale bar indicated on the movie) and various rotations angles to highlight the complex filamentous bacterial structures and the recruited neutrophils, that recognize and sense the growing bacteria, migrate to them, but fail to penetrate the yolk content, and to engulf the big bacterial aggregates. Due to the peculiar yolk composition and thickness, it was impossible to acquire the fluorescence of the bacteria growing inside the yolk distal to the objective, thus appearing as big dark spots.
(MP4)

S5 Movie *L*. *pneumophila* growing in the yolk region between 48 and 72 hpi: interactions with macrophages (related to Fig 3E). 4D, 40X objective, 6h time lapse between 48–72 hpi, 1microm optical sections, infected larva mounted ventral, bacteria growing in aggregate on the yolk. *Mfap4*: mCherry (red macrophages) 72hpf larva was injected in the bloodstream with HD of *L*. *pneumophila* WT-GFP, and was analyzed with confocal high microscopy between 48 and 72 hpi, to study the behavior of the bacteria growing in the yolk region, their spatial localization (above or below the yolk plasma membrane), and their interactions with macrophages overtime. The infected larva was mounted ventrally and acquired using a 40X water-immersion objective. Only the yolk region containing the bacterial aggregates was imaged. The acquired Z-stack was deconvolved using Leica Lightening Plug-in and processed for 4D visualization and volume rendering, using IMARIS 9.6 (Bitplane). Macrophage and bacteria surfacing automatically done, yolk region manually delimitated frame by frame with Imaris.
(MOV)

S6 Movie Macrophage—*L*. *pneumophila* interactions (LD, HD). *Mfap4*: mCherry (red macrophages) 72hpf larvae were injected in the bloodstream with LD (left panel) or HD (middle panel) of *L*. *pneumophila* WT-GFP or with HD OF *L*. *pneumophila* Δ*dotA-*GFP (right panel), mounted laterally and acquired using high resolution confocal microscopy to analyze macrophages (red cells) bacteria (green) interactions immediately upon bacteria injection. The infected larvae were acquired over time from 20 min to approximately 16 hours post injection. Maximum projections of the acquired Z-stacks are shown. The 3D movies generated were combined using Image J software, to have them side by side, to compare the macrophage-bacteria interaction over time in the various conditions. Left panel: *mfap4*: mCherry (red macrophages) 72hpf larva injected in the bloodstream with LD wt GFP *Legionella* (green). Note that macrophages are recruited to the injected bacteria, engulf them, and the bacteria are cleared progressively from the bloodstream. Middle panel: *mfap4*: mCherry (red macrophages) 72hpf larva injected in the bloodstream with HD wt GFP *Legionella* (green). Macrophages are recruited upon bacteria injection but failed to eliminate them over time; the phagocytosing macrophages round-up. Right panel: *mfap4*: mCherry (red macrophages) 72hpf larva injected in the bloodstream with HD GFP Δ*dotA Legionella* (green). Note that the recruited macrophages efficiently engulf and eliminate the injected bacteria, clearing them progressively from the blood and the mesenchyme near the point of injection.
(MP4)

S7 Movie Neutrophil—*L*. *pneumophila* (LD, HD) interactions. (*Lys*: DsRed (red neutrophils) 72hpf larvae were injected in the bloodstream with LD (left panel) or HD (middle panel) *L*. *pneumophila* WT-GFP, or with HD of Δ*dotA-*GFP (right panel), mounted laterally and acquired using high resolution confocal microscopy to analyze neutrophil (red cells) bacteria (green) interactions immediately upon bacteria injection. The infected larvae were acquired over time from 20 min to approximately 16 hours post injection. Maximum projections of the acquired Z-stacks are shown. The 3D movies generated were combined using ImageJ software, to have them side by side, to compare neutrophil-bacteria interactions over time in the various conditions. Left panel: *Lys*: DsRed (red neutrophils) 72hpf larva injected in the bloodstream with LD of WT-GFP (green). Note that neutrophils are recruited to the injected bacteria, engulfing the bacteria trapped in the mesenchyme near the site of injection, cooperating with macrophages (DsRed–cells, GFP+ having engulfed large amount of GFP bacteria), clearing progressively the infection. Middle panel: *Lys*:DsRed (red neutrophils) 72hpf larva injected in the bloodstream with HD of WT-GFP (green). Neutrophils are massively recruited upon bacterial injection but failed to eliminate them over time; the phagocytosing neutrophils round-up and loose DsRed fluorescence, suggesting cell death. Right panel: *lys*:DsRed (red neutrophils) 72hpf larva injected in the bloodstream with HD of Δ*dotA-*GFP *L*. *pneumophila* (green). Note that the recruited neutrophils engulf and eliminate the injected bacteria, clearing them progressively from mesenchyme near the point of injection, efficiently cooperating with macrophages in controlling the infection.
(MP4)

S8 Movie Dying phagocytosing neutrophils upon *L*. *pneumophila* HD injection (related to Fig 5). (*Lys*: DsRed (red neutrophils) 72hpf larvae were injected in the bloodstream with HD *L*. *pneumophila* WT-GFP, mounted laterally and acquired using high resolution confocal microscopy to analyze neutrophil (red cells) bacteria (green) interactions immediately upon bacteria injection. The infected larvae were acquired over time from 20 min to approximately 16 hours post injection. Time lapses every 1’30”. Maximum projections of the acquired Z-stacks (2mm per optical section) are shown. 6 neutrophils were manually tracked with Fiji (1 to 6) and highlighted with open white circle overtime. Note that the tracked neutrophils having engulfed *L*. *pneumophila* progressively dyed, rounding up and losing their red fluorescence, while the green fluorescence of the GFP bacteria is still visible overtime.
(MP4)
