# Supplementary figures and images for: Hiding in the yolk: A unique feature of Legionella pneumophila infection of zebrafish
Source: PLoS Pathog. 2023 May 8;19(5):e1011375. doi: 10.1371/journal.ppat.1011375 (PMC10194968; doi:10.1371/journal.ppat.1011375)

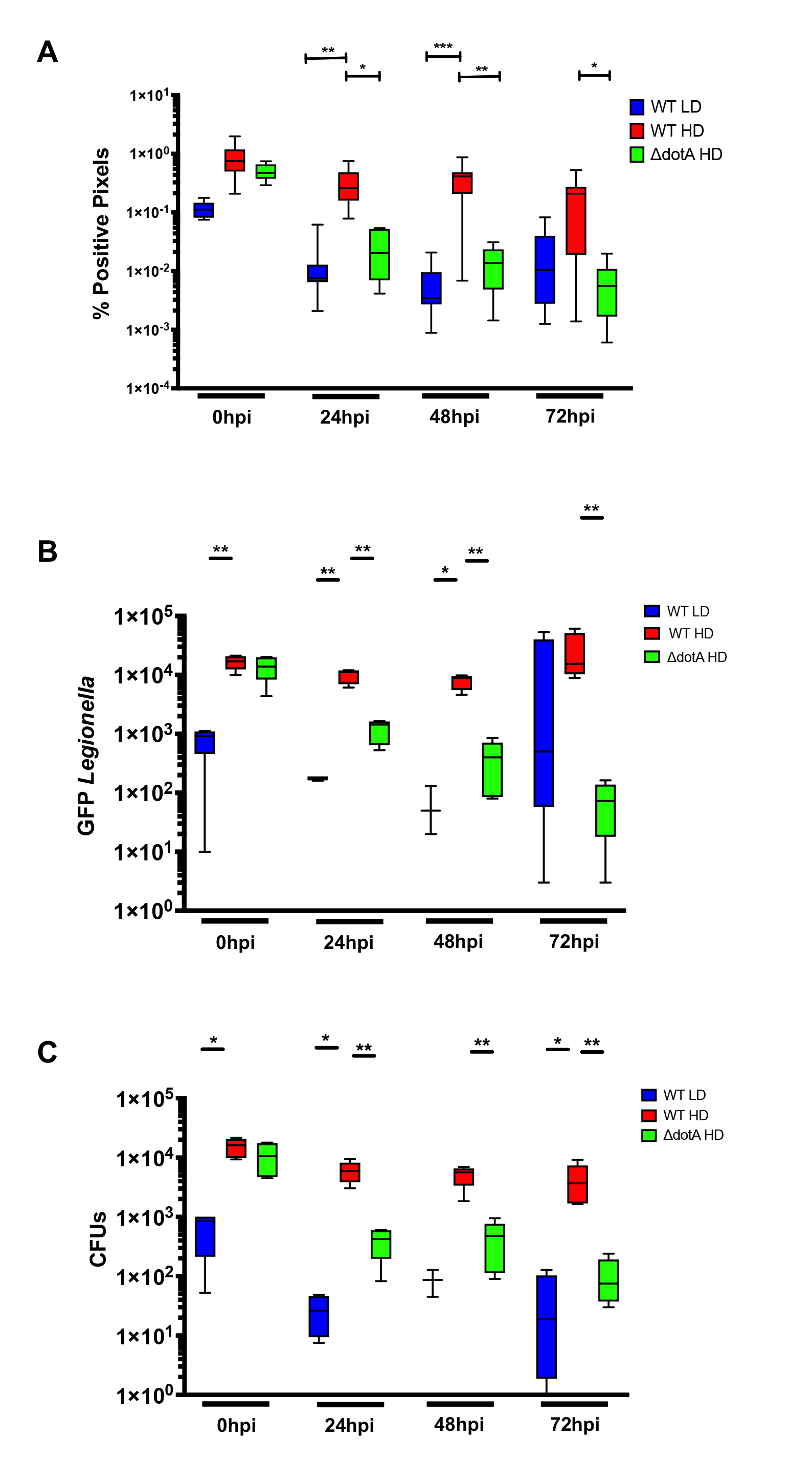

Supplement: S1 Fig — A) For bacterial burden measure by fluorescent pixel counts, the pictures corresponding to the GFP channel were analysed to quantify the percentage of fluorescent pixels using the ImageJ software. Individual larvae injected with WT-GFP Low Dose (LD) (blue symbols) or High Dose (HD) (red symbols) or infected with ΔdotA-GFP HD (green symbols) have been plotted and represented as box plot. Two independent experiments pooled, n = 10 larvae per condition). B) For FACS analyses, infected larvae were lysed and then GFP bacteria were counted on a MACSQuant VYB FACS (Miltenyi Biotec). One experiment plotted, n = 5 larvae per condition. C) CFUs were enumerated by plating serial dilutions of lysed infected larvae in BCYE agar supplemented with Chloramphenicol and Legionella Selective Supplement GVPN (Sigma). One experiment plotted, n = 5 larvae per condition. P < 0.05 was considered statistically significant (symbols: **** P < 0.0001; ***P < 0.001; **P < 0.01; *P < 0.05). No symbol on graphs means that not statistically differences were observed. (TIF) [file ppat.1011375.s001.tif]

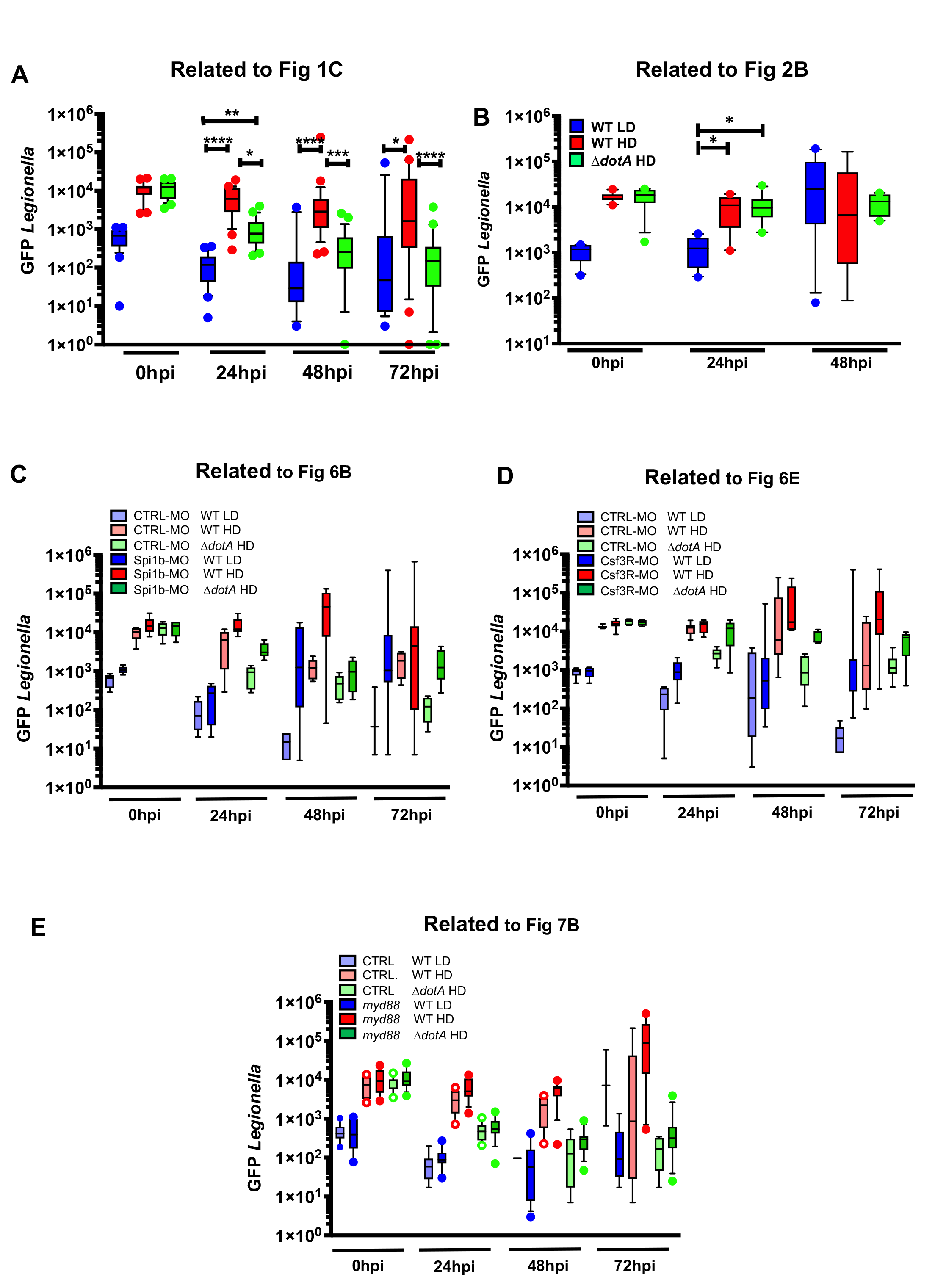

Supplement: S2 Fig — For FACS analyses, individual infected larvae were lysed and then GFP bacteria were counted on a MACSQuant VYB FACS (Miltenyi Biotec). A) Related to Fig 1C: 6 pooled experiments, n = 26 larvae for WT HD (28 for 72h), n = 26 larvae for WT LD (30 for 72h), n = 25 larvae for ΔdotA HD (26 for 72h) B) Related to Fig 2B: Fluorescent pixel count evaluation overtime upon yolk cell injection. One experiment is plotted. N = 6 larvae for WT HD, n = 6 larvae for WT LD, n = 5 larvae for ΔdotA HD. C) Related to Fig 6B: 2 pooled experiments, n = 8 Spi1b-MO larvae for WT HD, n = 8 Spi1b-MO larvae for WT LD, n = 5 Spi1b-MO larvae for ΔdotA HD, n = 5 control larvae for WT HD, n = 5 control larvae for WT LD and n = 5 control larvae for ΔdotA HD. D) Related to Fig 6E: 2 pooled experiments, n = 8 Csf3R-MO larvae for WT HD (10 for 72h), n = 8 Csf3R-MO larvae for WT LD (9 for 72h), n = 5 Csf3R-MO larvae for ΔdotA HD (4 for 0h), n = 6 control larvae for WT HD (8 for 72h), n = 6 control larvae for WT LD (9 for 72h) and n = 5 control larvae for ΔdotA HD (6 for 72h). E) Related to Fig 7B: 3 pooled experiments, n = 13 myd88 larvae for WT HD, n = 13 myd88 larvae for WT LD (12 for 0h), n = 13 myd88 larvae for ΔdotA HD, n = 10 control larvae for WT HD, n = 10 control larvae for WT LD, n = 10 control larvae for ΔdotA HD. P < 0.05 was considered statistically significant (symbols: **** P < 0.0001; ***P < 0.001; **P < 0.01; *P < 0.05). No symbol on graphs means that not statistically differences were observed. (TIF) [file ppat.1011375.s002.tif]

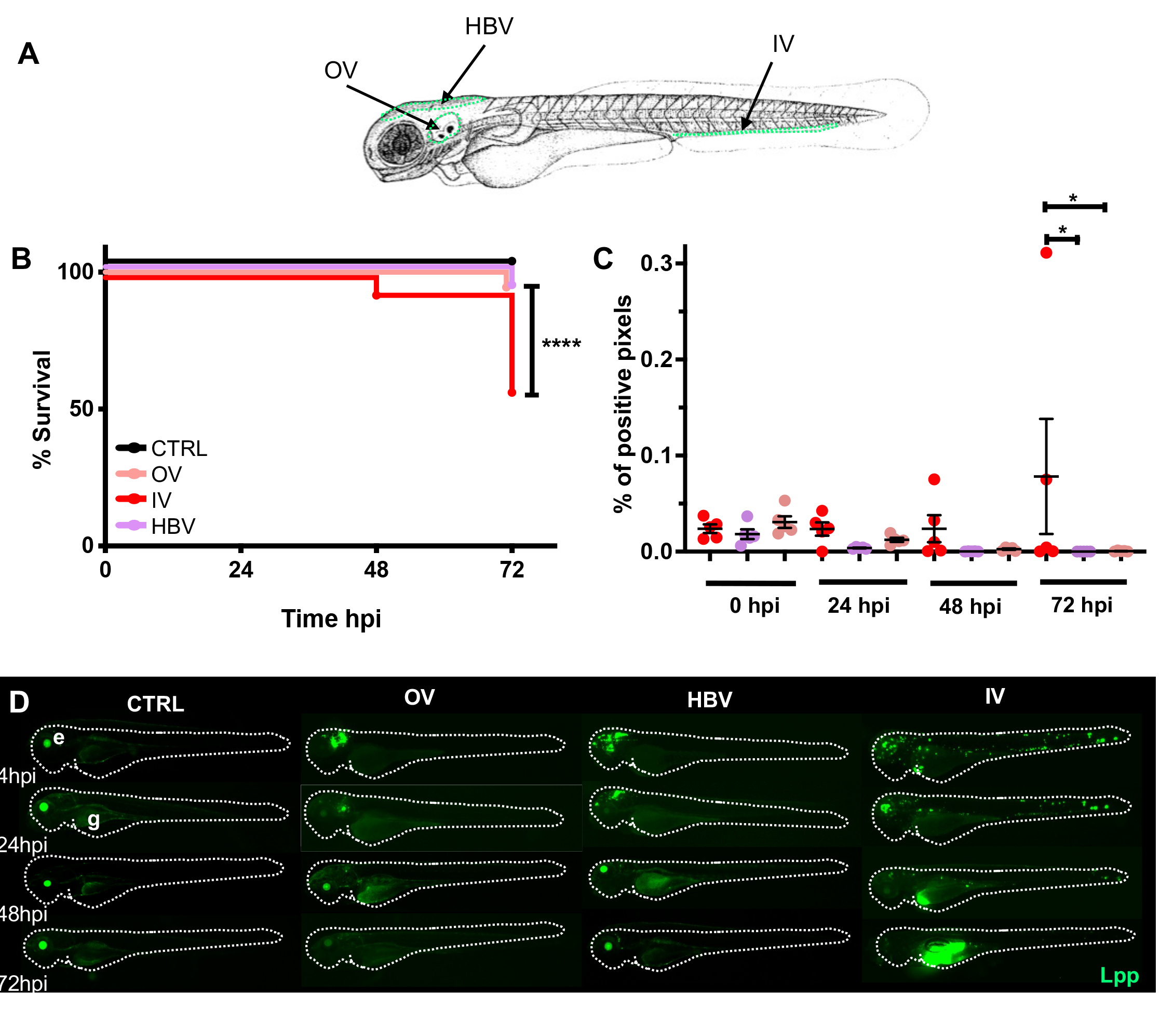

Supplement: S3 Fig — A) Scheme of 72hpf larva indicating the sites of bacterial injection. The scheme of the zebrafish larvae has been adapted from [35] and has been previously modified from [87]. Site of injection are indicated by green dashed boxes. OV: otic vesicle; HBV: hind brain ventricle; IV: intravenous injection. B. Survival curves. 2 experiments pooled; n = 36 larvae for CTRL and OV, 31 for IV, and 33 for HBV injection. C) bacterial burden evaluated over time by fluorescent pixel counts. 1 experiment, 6 larvae per condition. D. Representative images of L. pneumophila dissemination, determined by live imaging using a fluorescence stereomicroscope, of zebrafish larvae infected with a HD WT-, in closed compartments (OV, HBV) or in the bloodstream (IV). Infected larvae were live imaged 4h, 24h, 48h, and 72h post L. pneumophila injection. Only GFP fluorescence is shown. Green autofluorescence of the lens eye (e) or of the gastrointestinal tract (g) is indicated on CTRL larvae. P < 0.05 was considered statistically significant (symbols: **** P < 0.0001; ***P < 0.001; **P < 0.01; *P < 0.05). No symbol on graphs means that not statistically differences were observed. (TIF) [file ppat.1011375.s003.tif]

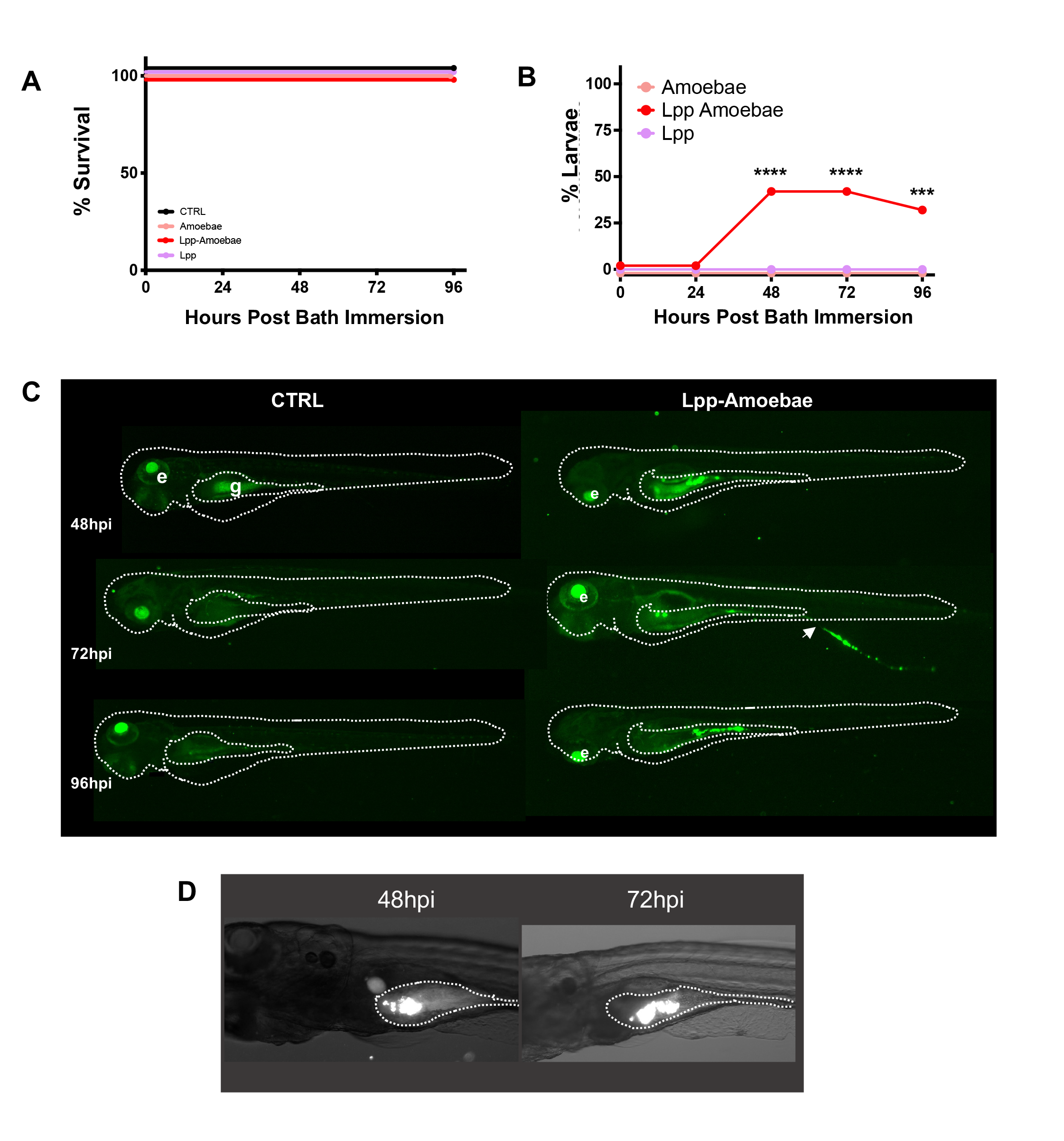

Supplement: S4 Fig — A) Survival curves. B) % of larvae with GFP bacteria. A and B: 1 experiment, 30 larvae for WT Lpp-amoebae, 10 for WT Lpp and 10 for amoebae. C) Representative fluorescent imaging of larvae with GFP bacteria in the intestinal tract followed over time. The intestinal tractus is highlighted with white dotted lines. Arrowhead points to GFP bacteria being eliminated with the fecal content. D) representative closeup of GFP bacteria in the intestinal tract. P < 0.05 was considered statistically significant (symbols: **** P < 0.0001; ***P < 0.001; **P < 0.01; *P < 0.05). No symbol on graphs means that not statistically differences were observed. (TIF) [file ppat.1011375.s004.tif]

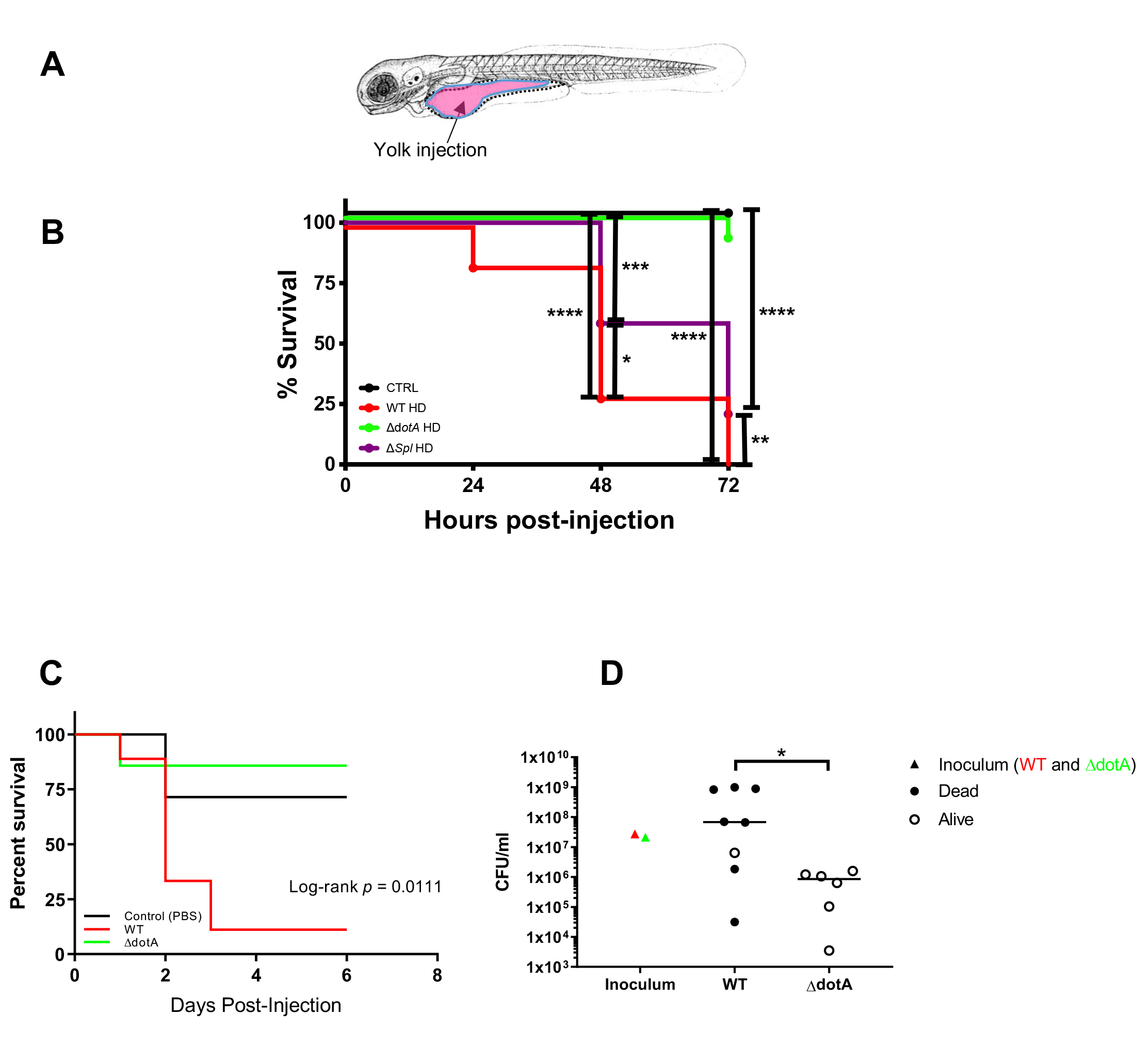

Supplement: S5 Fig — A). 72hpf larva: the yolk cell is highlighted in blue and the yolk content in pink. The scheme of the zebrafish larvae has been adapted from [35] and has been previously modified from [87]. B). Survival curves of 72hpf larvae upon injection in the yolk of HD WT (red curve), ΔdotA (green curve) or Δspi mutant (violet curve) L. pneumophila strain. CTRL larvae (black curve). One experiment, 24 larvae for each condition. Significant differences are indicated with stars. C) Survival curves of embryonated chicken eggs (ECE) inoculated with WT strain (red, n = 19 ECE in total corresponding to 9, 4, 3 and 3 ECE tested in the experiments No. 1, 2, 3 and 4 respectively), ΔdotA strain (green, n = 17 ECE in total corresponding to 8, 4, 2 and 3 ECE tested in the experiments No.1, 2, 3 and 4 respectively) or PBS (black, n = 17 ECE in total corresponding to 7, 4, 3 and 3 ECE tested in the experiments No.1, 2, 3 and 4 respectively). Survival is expressed in percentage and time in days. Comparison of survival curves was performed using Logrank (Mantel-Cox) test. P < 0.05 was considered statistically significant. D, E) Quantification of L. pneumophila (expressed in log10 CFU) in the yolk sac of WT-infected embryos (n = 19 in total) and ΔdotA infected embryos (n = 17 in total), according to the day of mortality of the embryos (D1, D2, D3 and alive at D6 (euthanized) (D) or the experiments (n = 4) (E). The inoculum after infection was estimated by considering the L. pneumophila count in the inoculum (WT and ΔdotA) before injection and the volume of the yolk sac. Comparison of the quantifications of L. pneumophila WT- or ΔdotA-infected embryos was done using the Mann-Whitney test. Medians and interquartile range are represented. P < 0.05 was considered statistically significant (**** P < 0.0001; ***P < 0.001; **P < 0.01; *P < 0.05). (TIF) [file ppat.1011375.s005.tif]

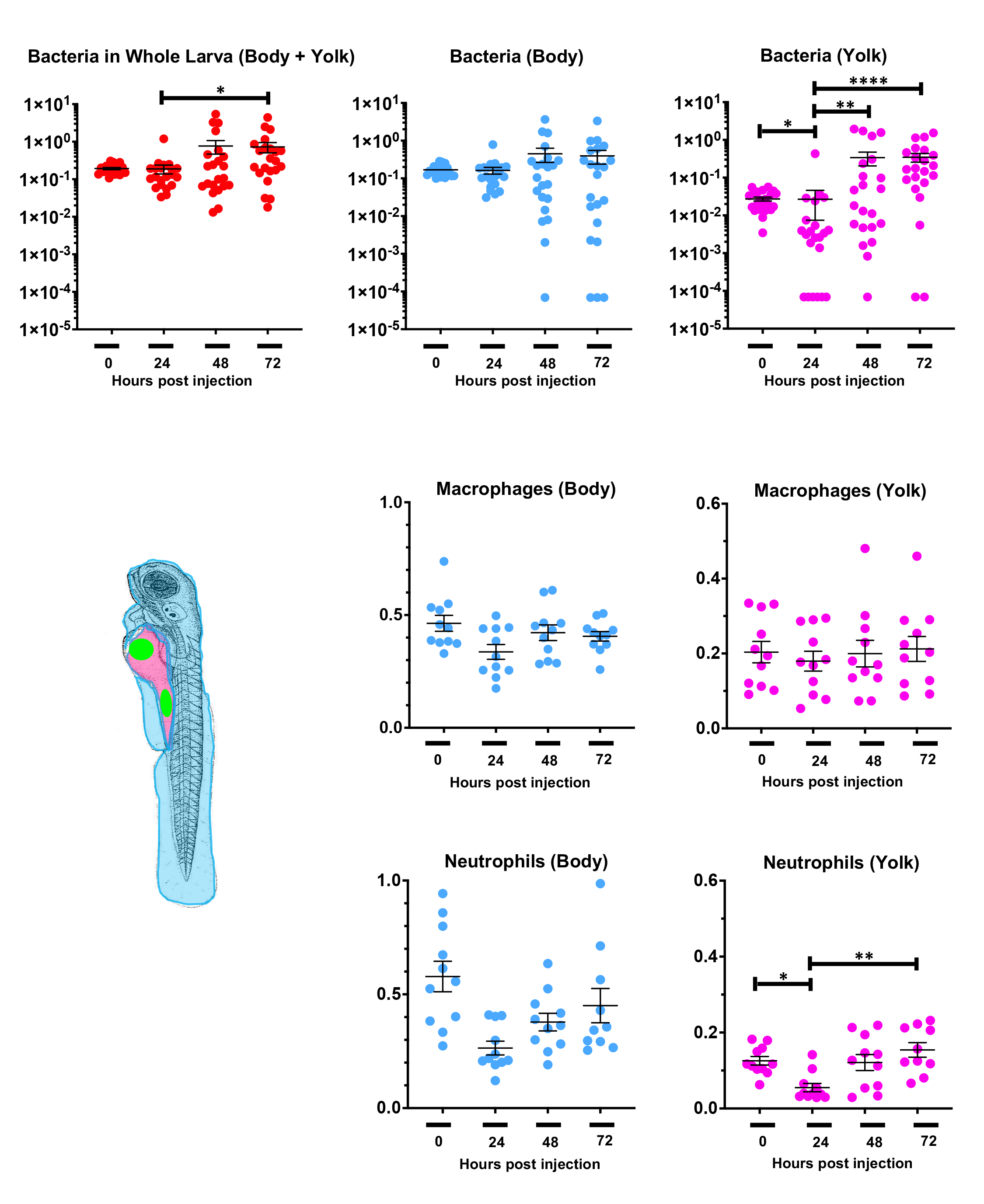

Supplement: S6 Fig — Two independent experiments plotted for each phagocyte type (total of 11 larvae for macrophage or 11 larvae for neutrophil quantification). Quantification of the fluorescent images (GFP bacteria and RFP leukocytes) was done using CellProfiler software (see Material and Methods for details about the pipeline). Bacterial burden quantification was done over the whole larva (red dot) or discriminating the body (light blue dot) form the yolk region (pink dot). Scheme of 72hpf with body (light blue) and yolk region (pink) highlighted; The scheme of the zebrafish larvae has been adapted from [35] and has been previously modified from [87]. The yolk sustaining L. pneumophila growing has been indicated with green dots. Quantification of macrophage or neutrophil located in body (light blue dot) or yolk (pink dot) over time. P < 0.05 was considered statistically significant (symbols: **** P < 0.0001; ***P < 0.001; **P < 0.01; *P < 0.05). No symbol on graphs means that not statistically differences were observed. (TIF) [file ppat.1011375.s006.tif]

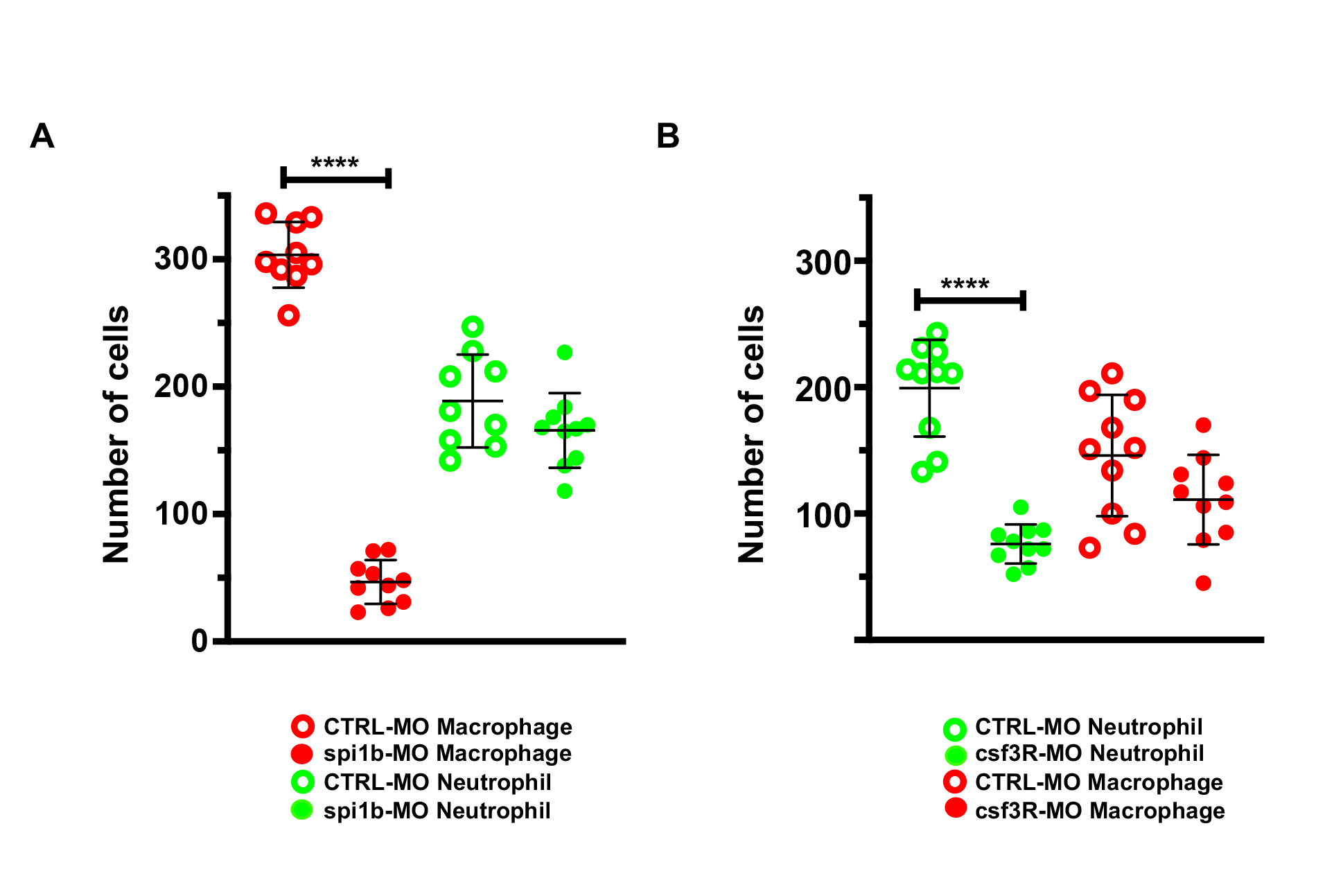

Supplement: S7 Fig — Comparison of the impact of spi1b morpholino injection that blocks macrophage development or csf3r morpholino injection that blocks neutrophil development were administered. Macrophages (red symbols) and neutrophils (green symbols) were counted in CTRL (open symbols) or morphant (full symbols) conditions. A) effect of spe1b morpholino on macrophages and neutrophils, showing that spe1b morpholino injection leads to the specific depletion of macrophages and not neutrophils. Related to Fig 4: 2 plotted experiments, n = 10 larvae per group. B) effect of Csf3R morpholino on macrophages and neutrophils, showing that Csf3R morpholino injection leads to the specific depletion neutrophils and slightly impairs the number of macrophages. Related to Fig 5: 2 plotted experiments, n = 10 larvae per group. (TIF) [file ppat.1011375.s007.tif]

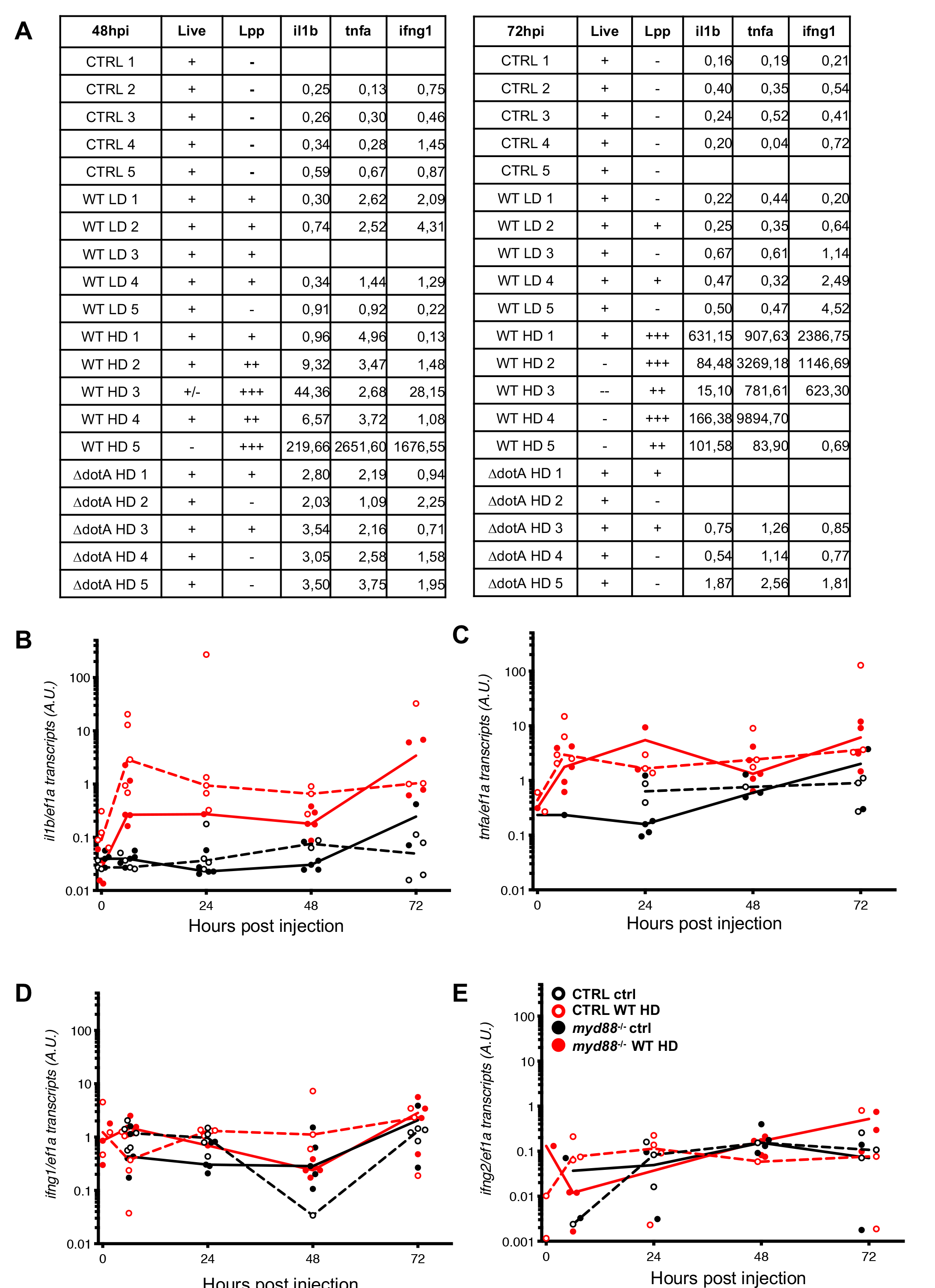

Supplement: S8 Fig — A. Correlation between bacterial burden (evaluated by fluorescence on individual injected larvae before RNA extraction) and cytokine gene induction at 48 and 72 hpi upon bloodstream injection LD, HD WT or HD ΔdotA L pneumophila strain. Control non injected, HD WT or HD ΔdotA injected larvae were scored under the fluorescent microscope for evaluating bacterial burden immediately before to be lysed and processed for RNA extraction. “-“, “+” to “+++ “respectively indicate no or in, creasing bacterial burden. “-”and “+” symbols were also used to respectively indicate infected dead or live larvae. Related to Fig 7A-7D. C-D) Cytokine gene (il1b, tnfa, ifng1/2) induction is independent from Myd88 signalling in L pneumophila HD WT infected zebrafish larvae.Cytokine gene induction was measured from individual myd88hu3568 mutant larvae injected with a HD (red curves) of WT-GFP and non-injected fish (CTRL, black curves). The same colours are used for individual CTRL non injected (black dashed) or HD WT injected (red dashed) zebrafish curves. Data plotted are from one experiment (n = 5 larvae for each condition); individual values are shown, and curves correspond to the medians. There is no statistically significant difference between CTRL and myd88hu3568 mutant curves over time for all the conditions analysed. Related to Fig 7E and 7F. (TIF) [file ppat.1011375.s008.tif]
